# Head-to-head evaluation of seven different seroassays including direct viral neutralisation in a representative cohort for SARS-CoV-2

**DOI:** 10.1099/jgv.0.001653

**Published:** 2021-10-08

**Authors:** Laura Olbrich, Noemi Castelletti, Yannik Schälte, Mercè Garí, Peter Pütz, Abhishek Bakuli, Michael Pritsch, Inge Kroidl, Elmar Saathoff, Jessica Michelle Guggenbuehl Noller, Volker Fingerle, Ronan Le Gleut, Leonard Gilberg, Isabel Brand, Philine Falk, Alisa Markgraf, Flora Deák, Friedrich Riess, Max Diefenbach, Tabea Eser, Franz Weinauer, Silke Martin, Ernst-Markus Quenzel, Marc Becker, Jürgen Durner, Philipp Girl, Katharina Müller, Katja Radon, Christiane Fuchs, Roman Wölfel, Jan Hasenauer, Michael Hoelscher, Andreas Wieser, on behalf of the KoCo19-Study Group

**Affiliations:** ^1^​ Division of Infectious Diseases and Tropical Medicine, University Hospital, LMU Munich, 80802 Munich, Germany; ^2^​ German Center for Infection Research (DZIF), Partner site Munich, Germany; ^3^​ Institute of Radiation Medicine, Helmholtz Zentrum München, 85764 Neuherberg, Germany; ^4^​ Institute of Computational Biology, Helmholtz Zentrum München, 85764 Neuherberg, Germany; ^5^​ Center for Mathematics, Technische Universität München, 85748 Garching, Germany; ^6^​ Department of Business Administration and Economics, Bielefeld University, 33615 Bielefeld, Germany; ^7^​ Bavarian Health and Food Safety Authority (LGL), Germany; ^8^​ Core Facility Statistical Consulting, Helmholtz Zentrum München, 85764 Neuherberg, Germany; ^9^​ BRK-Blutspendedienst, 80336 Munich, Germany; ^10^​ Department of Conservative Dentistry and Periodontology, University Hospital, LMU Munich Ludwig-Maximilians-University of Munich, Goethestr. 70, 80336 Munich, Germany; ^11^​ Laboratory Becker and colleagues, Führichstr. 70, 81671 München, Germany; ^12^​ Bundeswehr Institute of Microbiology, 80937 Munich, Germany; ^13^​ Institute and Outpatient Clinic for Occupational, Social and Environmental Medicine, University Hospital, LMU Munich, 80336 Munich, Germany; ^14^​ Center for International Health (CIH), University Hospital, LMU Munich, 80336 Munich, Germany; ^15^​ Comprehensive Pneumology Center (CPC) Munich, German Center for Lung Research (DZL), 80337 Munich, Germany; ^16^​ Faculty of Mathematics and Natural Sciences, University of Bonn, 53113 Bonn, Germany

**Keywords:** antibody, COVID-19, nucleocapsid, RBD, SARS-CoV-2, serology, spike, virus neutralisation

## Abstract

A number of seroassays are available for SARS-CoV-2 testing; yet, head-to-head evaluations of different testing principles are limited, especially using raw values rather than categorical data. In addition, identifying correlates of protection is of utmost importance, and comparisons of available testing systems with functional assays, such as direct viral neutralisation, are needed.We analysed 6658 samples consisting of true-positives (*n*=193), true-negatives (*n*=1091), and specimens of unknown status (*n*=5374). For primary testing, we used Euroimmun-Anti-SARS-CoV-2-ELISA-IgA/IgG and Roche-Elecsys-Anti-SARS-CoV-2. Subsequently virus-neutralisation, GeneScriptcPass, VIRAMED-SARS-CoV-2-ViraChip, and Mikrogen-*recom*Line-SARS-CoV-2-IgG were applied for confirmatory testing. Statistical modelling generated optimised assay cut-off thresholds. Sensitivity of Euroimmun-anti-S1-IgA was 64.8%, specificity 93.3% (manufacturer’s cut-off); for Euroimmun-anti-S1-IgG, sensitivity was 77.2/79.8% (manufacturer’s/optimised cut-offs), specificity 98.0/97.8%; Roche-anti-N sensitivity was 85.5/88.6%, specificity 99.8/99.7%. In true-positives, mean and median Euroimmun-anti-S1-IgA and -IgG titres decreased 30/90 days after RT-PCR-positivity, Roche-anti-N titres decreased significantly later. Virus-neutralisation was 80.6% sensitive, 100.0% specific (≥1:5 dilution). Neutralisation surrogate tests (GeneScriptcPass, Mikrogen-*recom*Line-RBD) were >94.9% sensitive and >98.1% specific. Optimised cut-offs improved test performances of several tests. Confirmatory testing with virus-neutralisation might be complemented with GeneScriptcPass^TM^ or *recom*Line-RBD for certain applications. Head-to-head comparisons given here aim to contribute to the refinement of testing strategies for individual and public health use.

## Impact statement

We present an evaluation of seven serological SARS-CoV-2 tests used as screening or confirmation tests in a large, well-defined cohort including true-positive and true-negative individuals, as well as subjects with unknown SARS-CoV-2 status. A total of 6658 individual samples were derived from the Representative COVID-19 Cohort Munich (KoCo19), a prospective seroincidence study initiated in Munich, Germany, in a low-prevalence setting of a large German city, not considered a specific ‘hot-spot’ at time of sampling. This comprehensive collection allowed us to correlate the different tests to identify concordance as well as discordance for the individual samples. This also enabled the evaluation of confirmatory test systems, like direct virus-neutralisation or the recently FDA-approved GeneScriptcPassTM, compared to different serological tests, including receptor binding domain (RBD)-based assays. In addition, we assessed their overall performances in KoCo19, and adjusted cut-offs; furthermore, we analysed the seroconversion rate in patients with a history of positive RT-PCR.

## Introduction

In December 2019, a cluster of atypical pneumonia of unknown origin was described in the region of Wuhan, Hubei province, China. Subsequently, a previously unknown coronavirus was identified as the causative agent: SARS-CoV-2 (Severe Acute Respiratory Syndrome Coronavirus 2) [1]. As the virus spread rapidly across the globe, the Corona Virus Disease 2019 (COVID-19) was declared a pandemic on 12 March 2020.

Direct detection of viral nucleic acids or the virus itself in bodily fluids is considered the reference standard for diagnosis of acute infection. It is primarily performed using nasopharyngeal swabs or other respiratory samples [2]. Additionally, serodiagnostics are valuable to identify past infections, asymptomatic or symptomatic, and to elucidate transmission dynamics within populations. Both are highly relevant to inform evidence-based political decision making [3, 4].

Several serological test systems have been introduced since the beginning of the SARS-CoV-2 pandemic [5]. Most target one of two specific viral structures: parts of the trimeric CoV spike (S1-2) complex, or the nucleocapsid (N) protein [6]. While the receptor binding domain (RBD) of S1 binds to the angiotensin-converting enzyme 2 (ACE2) as a receptor, the N-protein is involved in viral assembly and replication [7]. Head-to-head comparisons evaluating qualitative assay performances have been described, yet mostly with limited additional workup and sample characterisation [8–11]. Some authors have proposed adapted cut-off thresholds to increase assay performance, depending on application and local epidemiology [4, 12].

Here, we present a head-to-head cross-comparison of seven independent tests. We screened with Euroimmun Anti-S1-SARS-CoV-2-ELISA-IgA and -IgG and Elecsys Anti-SARS-CoV-2 Roche N pan−Ig and confirmed with direct viral neutralisation, GeneScriptcPass, Mikrogen-*recom*Line-RBD IgG line immunoassay, and VIRAMED-SARS-CoV-2-ViraChip microarray. The tests were conducted on a total of 6658 samples from (i.) RT-PCR positive individuals (true-positives), (ii.) blood donors from the pre-COVID-19 era (true-negatives), and (iii.) subjects with unknown disease status from a representative population cohort in Munich (KoCo19; unknown serostatus) [13]. We were able to generate reliable performance estimates for both primary and confirmatory tests by using true-positive and true-negative individuals and hereby generate optimised cut-offs.

## Methods

### Study design and participants

Samples are derived from the representative COVID-19 Cohort Munich (KoCo19), a prospective sero-incidence study initiated in Munich, Germany, in April 2020 [13, 14]. For this study, we tested 6658 samples, including a set of SARS-CoV-2 RT-PCR positives (‘true-positives’, *n*=193), individuals from historical cohorts, blood donors without any indication of SARS-CoV-2 infection (‘true-negatives’, *n*=1091), and specimen of unknown status (*n*=5374); details on the cohort characteristics, including collection time points, can be found in the appendix (p.1; Table S1, available in the online version of this article).

The study was approved by the Ethics Committee of the Faculty of Medicine at LMU Munich (20–275 V), the protocol is available online (www.koco19.de) [13]. Informed consent was obtained prior to any study investigations where applicable. The study is registered in the German Clinical Trials Register (DRKS00021698;https://www.drks.de/drks_web/navigate.do?navigationId=trial.HTML&TRIAL_ID=DRKS00021698).

### Laboratory assays

All described analyses were performed using EDTA-plasma samples (appendix pp.1 for further details on assays performed, and Table S3 for details on platforms and units applied).

Euroimmun Anti-SARS-CoV-2-ELISA anti-S1 IgA/IgG (called EI-S1-IgG, EI-S1-IgA; Euroimmun, Lübeck, Germany) test kits were used according to the manufacturer’s instructions. Measurement values were obtained using the quotient of the optical density measurement provided by the manufacturer’s software. We evaluated Elecsys Anti-SARS-CoV-2 Roche anti-N pan-Ig (hereafter called Ro-N-Ig; Roche, Mannheim, Germany) in accordance with the manufacturer’s guidelines. Values reported are the Cut-Off-Index (COI) of the individual samples. Operative replicates of the same samples were performed to assess reliability of primary assay performance.

For confirmatory testing, we conducted micro-virus neutralisation assays (NT) as described previously [15], with the exception that confluent cells were incubated instead of adding cells following neutralisation reaction (appendix pp.1). We classified samples with a titre <1:5 as ‘NT-negative’ and samples with a titre ≥1:5 as ‘NT-positive’. The dilution steps indicated are <5, 5, 10, 20, 40 and >80.

SARS-CoV-2 surrogate virus neutralisation test (GS-cPass; GenScript, Piscataway, New Jersey, USA) was used to measure binding inhibition, according to the manufacturer’s instructions. The inhibition was calculated in percentages, ranging from −30 to 100.

For SARS-CoV-2 ViraChip microarray (VIRAMED Biotech AG, Planegg, Germany; hereafter named VC-N-IgA/IgM/IgG; VC-S1-IgA/IgM/IgG; VC-S2-IgA/IgM/IgG) execution followed the manufacturer’s instructions. We obtained measurement values by the automated ELISA-processor in arbitrary units.

We conducted the *recom*Line SARS-CoV-2 IgG line immunoassay (MG-S1, MG-N, MG-RBD; Mikrogen, Neuried, Germany) as outlined by the manufacturer. Values below the cut-off of 1 were categorised as ‘negative’ without quantitative information.

### Statistical analysis

Prior to analysis, we cleaned and locked the data. For the analyses and visualisation, we used the software R, version 4.0.2. Only one sample per subject was included in the statistical analyses; in the case of individuals with multiple blood samples, we only considered the sample with the most complete dataset. For multiple measurements with complete datasets, we only included the first measurement; for operational replicates we used the latest one. We subsequently carried out sensitivity and specificity analyses for true-negative and true-positive samples over all the tests performed.

We report square roots R of coefficients of determination for association among continuous variables. For paired sample comparisons, we applied Wilcoxon-sign-rank tests, whereas for multiple group comparisons we applied Kruskal-Wallis tests, followed by post-hoc Dunn tests using the Benjamini-Yekutieli adjustment for pairwise comparisons [16].

Using true-positives and true-negatives, we determined optimised cut-off thresholds and their confidence intervals by a nonparametric bootstrap. In a similar way, we trained random forest and support vector machine classifiers. We calculated estimates for sensitivities, specificities, and overall prediction accuracies for all considered cut-off values and classifiers. This calculation was done on out-of-sample observations to avoid overfitting and thus overoptimistic performance measures. Details on the algorithms are outlined in the appendix (pp.3).

### Data and code sharing

Data are accessible subject to data protection regulations upon reasonable request to the corresponding author. To facilitate reproducibility and reuse, the code used to perform the analyses and generate the figures was made available on GitHub (https://github.com/koco19/lab_epi) and has been uploaded to ZENODO (is https://doi.org/10.5281/zenodo.4699432) for long-term storage.

## Results

We assessed SARS-CoV-2 antibodies in a total of 6658 independent samples using Euroimmun Anti-SARS-CoV-2-ELISA anti-S1 IgA (henceforth called EI-S1-IgA; *n*=6657), Euroimmun Anti-SARS-CoV-2-ELISA anti-S1 IgG (EI-S1-IgG; *n*=6658), and Elecsys Anti-SARS-CoV-2 Roche anti-N pan-Ig (Ro-N-Ig; *n*=6636) (details on cohort and sample characteristics are outlined in Fig. S1, Table S1). Sensitivity and specificity estimates of both primary and confirmatory tests of manufacturer and optimised cut-offs are shown in Tables 1 and S2 features an overview of all tests performed.

**Table 1.** Manufacturer's and optimised cut-offs, sensitivity, specificity and accuracy Evaluation of diagnostic accuracy of primary tests was conducted with samples from true-positives (*n*=193) and true-negatives (*n*=1073); subsequently, optimised cut-offs were applied to the KoCo19-cohort samples (see Methods).

Sample composition True pos. / true neg.	Test	Manuf.’s cut-off	Optimised cut-off [CI]	Sensitivity [%] (Manuf.’s / Optim. cut-off)	Specificity [%] (Manuf.’s / Optim. cut-off)	Overall accuracy [%](Manuf.’s / Optim. cut-off)
193/1073	EI-S1-IgA	1.100	1.085 [0.855; 1.705]	64.77/64.77	93.29/92.64	88.94/88.39
193/1073	EI-S1-IgG	1.100	1.015 [0.850; 1.395]	77.20/79.79	98.04/97.76	94.87/95.02
193/1073	Ro-N-Ig	1.000	0.422 [0.295; 0.527]	85.49/88.60	99.81/99.72	97.63/98.03
107/106	NT	–	5.0*	- / 73.83	- / 100.00	- / 86.85
108/106	GS-cPass	20.000	20.538 [13.768; 24.241]	96.30/96.30	100.00/99.06	98.13/97.66
108/111	VC-N-IgG	100.000	18.500 [13.500; 23.000]	39.81/93.52	99.10/91.89	69.86/92.69
108/111	VC-S1-IgG	100.000	10.000 [10.000; 10.000]	65.74/95.37	100.00/100.00	83.11/97.72
108/111	VC-S2-IgG	100.000	10.000 [10.000; 10.000]	17.59/63.89	100.00/99.10	59.36/81.74
78/106	MG-N	1.000	1.000 [1.000; 1.600]	94.87/94.87	98.11/98.11	96.74/96.74
78/106	MG-RBD	1.000	1.000 [1.000; 1.000]	94.87/94.87	100.00/100.00	97.83/97.83
78/106	MG-S1	1.000	1.000 [1.000; 1.000]	96.15/96.15	100.00/100.00	98.37/98.37
193/1073	Random Forest	–	–	88.60†	99.81†	98.10†
193/1073	Support Vector Machine	–	–	84.46†	99.91†	97.47†

*For NT, dilutions starting at 1:5 were used; see Methods.

†The random forest and the support vector machine combine all three primary tests, the accuracy measures thus do not relate to specific cut-offs.

### Performance of primary tests

**Fig. 1.**
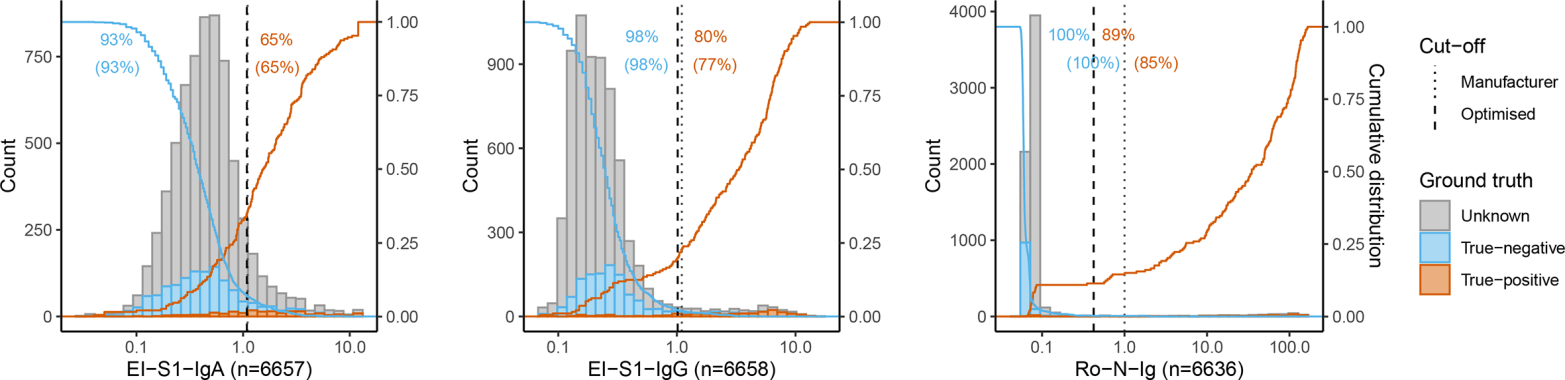
Performance of primary tests. Results of primary tests for true-negatives (*blue*), true-positives (*orange*), and individuals with unknown SARS-CoV-2 status (*grey*). Absolute number of subjects (count/*y-axis*) and distribution of raw values (*x-axis*) measured for EI-S1-IgA (*left*), EI-S1-IgG (*centre*), and Ro-N-Ig (*right*). Dotted lines mark the manufacturer’s cut-off value (between indeterminate and positive for EI, and between negative and positive in Ro). Dashed lines mark the optimised cut-off value as determined in this study (overlapping with the dotted line for EI-S1-IgA). Orange and blue solid lines represent the percentage of test results for true-positives and true-negatives above (*blue*) or below (*orange*) the value on the x-axes, respectively. Orange and blue numbers give the percentages of true-positives and true-negatives that were correctly detected by the test (within brackets: manufacturers' cut-offs; without brackets: optimised cut-offs).

Sensitivity and specificity of EI-S1-IgA were 64.8 and 93.3% when applying the manufacturer’s cut-off. Optimising the cut-off through statistical learning (see Methods) did not improve EI-S1-IgA performance (sensitivity 64.8%, specificity 92.6%). For EI-S1-IgG, the sensitivity of 77.2 % (manufacturer’s cut-off) was increased to 79.8% (optimised cut-off), while the specificity remained similar at 98.0/97.8 % (manufacturer’s/optimised cut-off; Table 1). The distribution of results for the EI-assays is depicted in Fig. 1. Raw values for EI-S1-IgA show a slightly asymmetric but unimodal distribution for the overall population, while EI-S1-IgG raw values present with a second clearly distinct positive population. EI-S1-IgA classified 65% of the true-positives correctly as positive and 7% of the true-negatives incorrectly as positive, while EI-S1-IgG classified 80% of the true-positives correctly and 2% of the true-negatives incorrectly. A total of 61% of the true-positives were identified correctly by both tests unanimously.

The sensitivity of Ro-N-Ig with the manufacturer’s cut-off was 85.5%, and was increased to 88.6% by applying an optimised cut-off, similarly to EI-S1-IgG. Specificity was similar with both cut-offs at 99.8/99.7 % (manufacturer’s/optimised cut-off; Table 1). Ro-N-Ig raw values (Cut-off index, COI) demonstrate a narrow distribution with the bulk of values in the range COI 0.1 and below, whereas a clearly separated second population above COI 10 was observed. For EI-S1-IgG and Ro-N-Ig, the cut-offs separate the subpopulations more reliably than for EI-S1-IgA (Fig. 1).

**Fig. 2.**
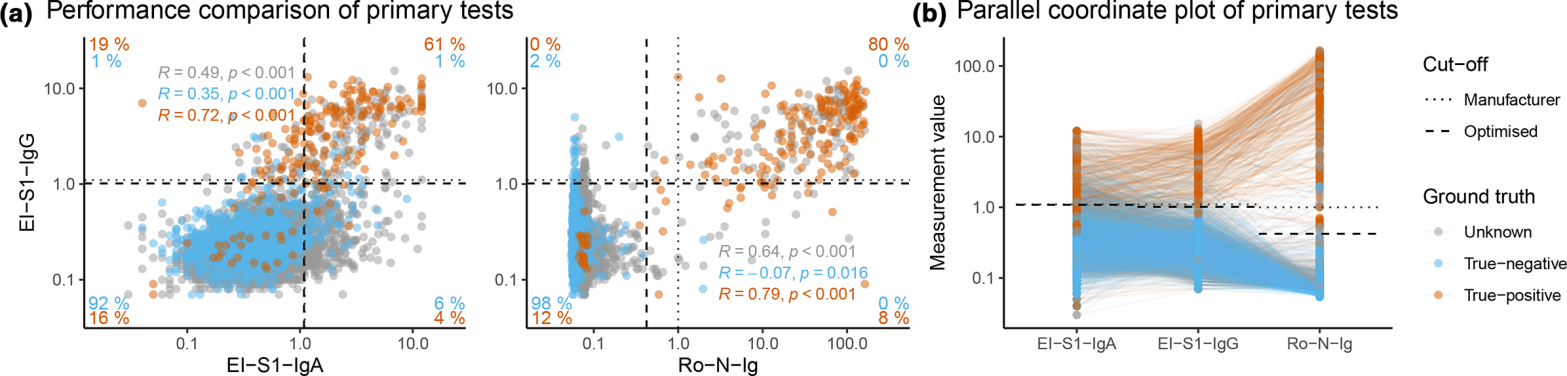
Comparison of primary tests. Results of primary tests compared to ground truth for true-negatives (*blue*), true-positives (*orange*), and individuals with unknown SARS-CoV-2 status (*grey*). The dotted lines represent the manufacturer’s cut-offs, the dashed lines the optimised cut-offs defined within this study. (**a**) Pairwise scatter plots for EI-S1-IgA vs. EI-S1-IgG (left; *n*=6657), and Ro-N-Ig vs. EI-S1-IgG (right; *n*=6636). Percentages in orange indicate fractions of true-positives in the respective quadrant with respect to all true-positives; blue for true-negatives. Percentages were calculated using the optimised cut-off. (**b**) Parallel coordinate plot of the same three tests.

For evaluation of primary test concordance, we excluded EI-S1-IgA due to inferior performance in sensitivity and specificity. The concordance between EI-S1-IgG and Ro-N-Ig was 98.5% (6538/6636). From the whole sample set, 4.0% (264/6636) were unanimously classified as positive while 94.5% (6274/6636) were classified as negative, of these 88.1% (5846/6635) tested negative in all three tests (being 93.2% (5846/6274) of those negative in Ro-N-Ig+EI-S1-IgG). The remaining 1.5% of samples (98/6636) were classified discordantly. Of these, 56.1% (55/98) were rated as positive by El-S1-IgG and as negative by Ro-N-Ig (Fig. 2a), while the remaining 43.9% (43/98) were rated as negative by EI-S1-IgG and as positive by Ro-N-Ig.

**Fig. 3.**
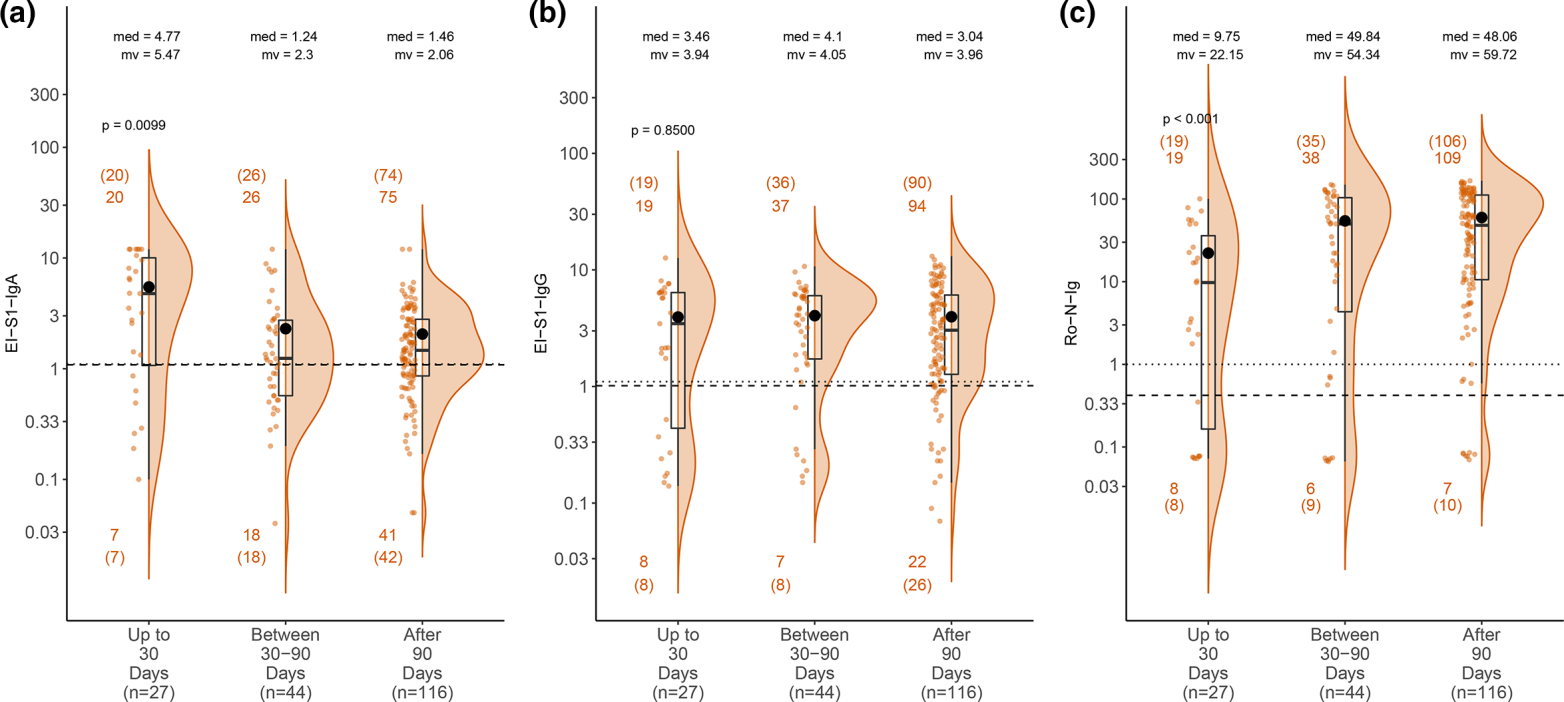
Time dependence in primary tests for RT-PCR true-positives. Titre values of 187 true-positives with available data on time between RT-PCR and blood sampling for (**a**) EI-S1-IgA, (**b**) EI-S1-IgG, and (**c**) Ro-N-Ig. The read-outs were categorised according to the time after positive RT-PCR (<30 days, 30–90 days and >90 days). Plots show the individual read-out (orange dots), a density estimate (orange area), the 25-,50- and 75-percentiles (black boxes), and the means (black dots). Counts *n* (*n* refer to the number of observations above/below manufacturer’s (optimised) cut-off for each of the temporal groups). Pairwise differences are considered only after adjusting for multiple testing and can be found in Table S4. Mean values (mv) and median values (med) are given for each group.

We investigated seropositivity following positive RT-PCR using one measurement per subject (Fig. 3). EI-S1-IgA titres were found to decline >30 days (*P*=0.01), with only 65% being positive at the last interval, while EI-S1-IgG remained stable over the time period (*P*=0.85). In contrast, antibody levels measured with Ro-N-Ig increased over time (*P*<0.001).

### Performance of confirmatory tests

**Fig. 4.**
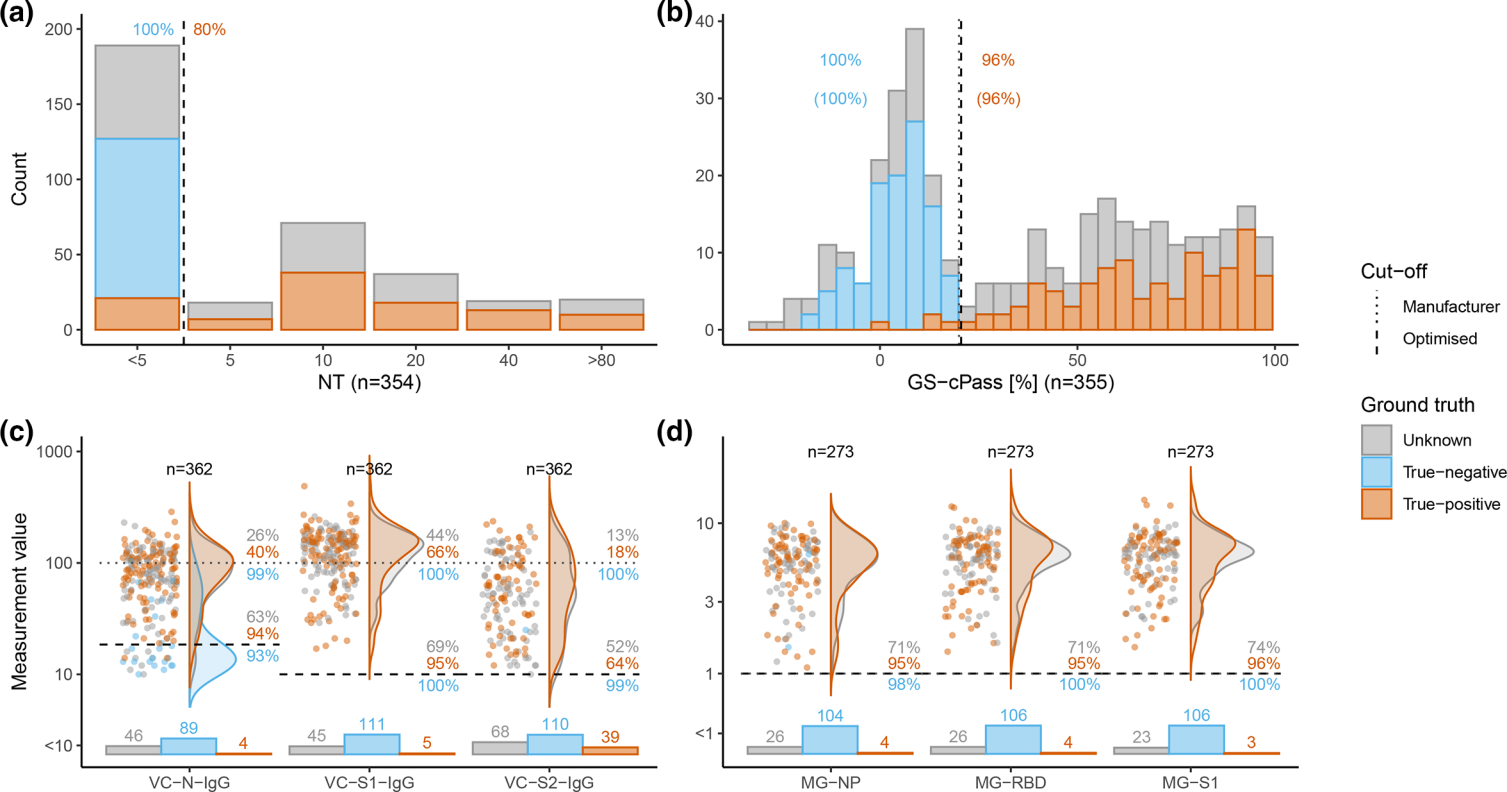
Confirmatory tests. Results of confirmatory tests compared to ground truth for true-negatives (*blue*), true-positives (*orange*), and individuals with unknown SARS-CoV-2 status (*grey*). Black dotted and dashed lines represent the manufacturers and the optimised cut-offs, respectively. Orange/blue numbers indicate percentages of true-positives/-negatives correctly detected by the test using the respective cut-offs (identical in **a**, **b, d**). Distribution of results of NT (**a**) and GS-cPass (**b**). Distribution of IgG results of the VC-array (**c**) and the MG-line blot (**d**). Bar charts below violin plots represent information on the categorical part of the values below linear range. Grey numbers give the percentages of positive samples with unknown SARS-CoV-2 as determined by the manufacturers and optimised cut-offs. Percentages were calculated over the total number of samples of unknown SARS-CoV-2 status with available test results.

We subjected a sample subset (*n*=362; composition see Fig. S1) to confirmatory testing; the overall confirmatory test performance is presented in Fig. 4 and Table 1. The sensitivity of direct neutralisation titres (NT; 1:5 dilution) was 80.6%, 96.3% for GS-cPass, and 94.9 % for MG-RBD. All three tests had a specificity close to 100 % (Fig. 4). Adjustments of the cut-off in these three systems did not improve the performance (shown in parentheses in the Figures). NT-titres in our cohort were low – mostly 1:5 – and only few subjects had high NT of 1 : 80 or above (Fig. 4a).

For the VC-array, sensitivities of both VC-S1-IgG and VC-N-IgG were improved markedly by optimising cut-offs, with gains of >30% (VC-N-IgG 39.8/93.5%; VC-S1-IgG 65.7/95.4%; see Table 1, Fig. 4c). Performance of VC-S2-IgA and VC-S2-IgM are presented for reference in Fig. S5.

**Fig. 5.**
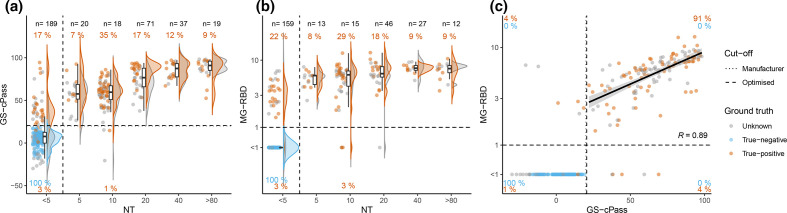
Comparison of confirmatory tests. Comparison of confirmatory tests for true-negatives (*blue*), true-positives (*orange*), and individuals with unknown SARS-CoV-2 status (*grey*). At the top, in black, total number of cases (n) for each NT category. (**a**) Association between the categorical endpoint of NT and the continuous results of GS-cPass (*n*=354). (**b**) Association between the categorical endpoint of NT and the continuous results of MG-RBD (*n*=272). (**c**) Association between GS-cPass and MG-RBD (*n*=272). The solid black line represents a linear regression for the positive measurements.

The categorical endpoints of NT and the continuous results of GS-cPass were positively related (R2=0.74), agreement with the ground truth was frequent (80%). However, more than 17% of true-positive samples were negative in NT (*n*=21, Fig. 5a). Correlation between NT and MG-RBD was similar to GS-cPass (*n*=272, Fig. 5b). However, separation between the negative and positive population was better in MG-RBD than with GS-cPass, especially in those true-positives with low direct neutralisation capacity (NT <5). Association between GS-cPass and MG-RBD was good (*n*=272, Fig. 5c), discordant results were observed in 8% of true-positives. The distribution presented as increasingly narrower in higher titre ranges.

### Associations of confirmatory and primary tests

**Fig. 6.**
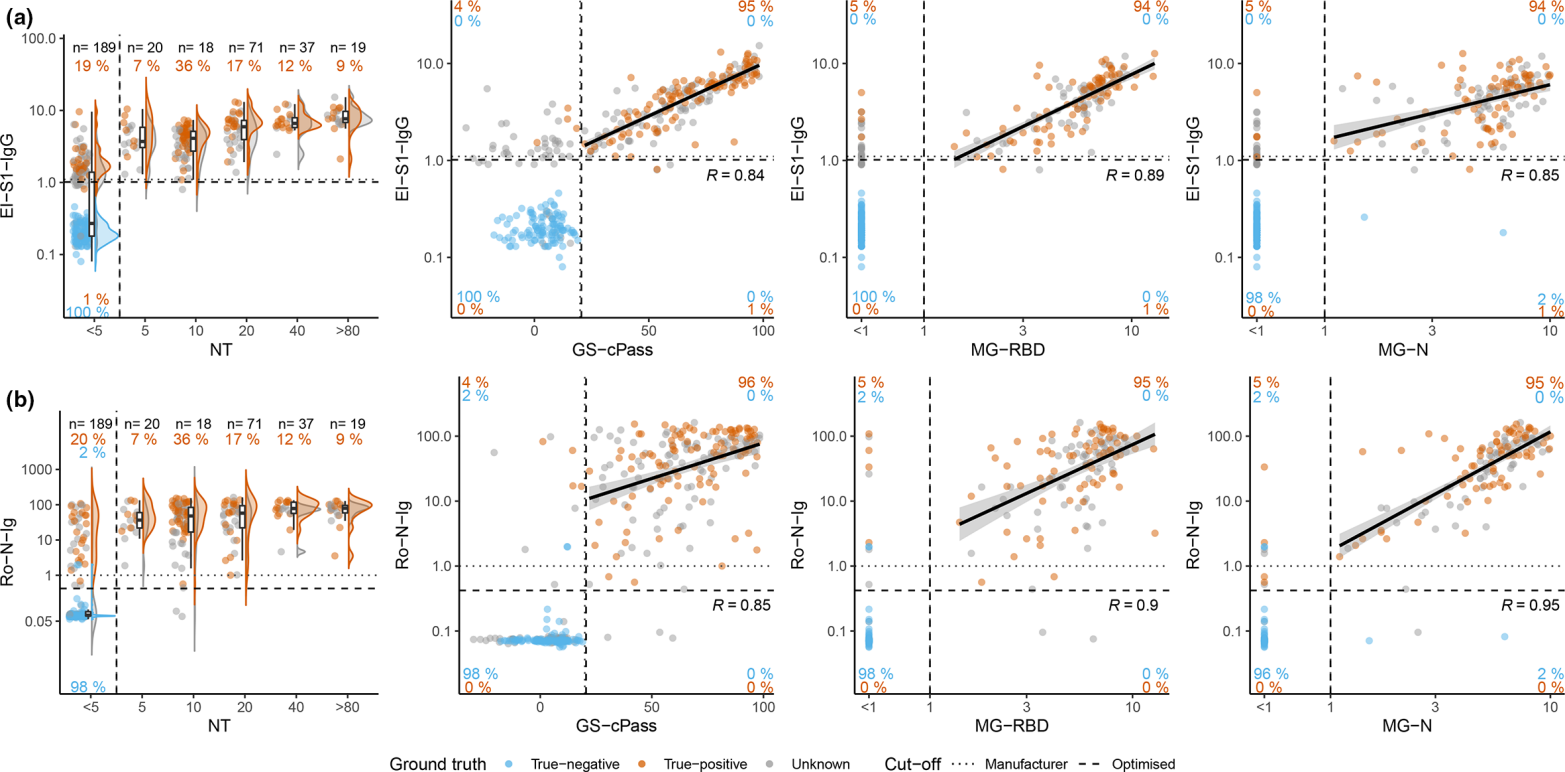
Comparison of primary tests (EI-S1-IgG, Ro-N-Ig) with confirmatory tests (NT, GS-cPass MG-RBD, MG-N). Comparison of EI-S1-IgG and Ro-N-Ig with confirmatory tests for true-negatives (*blue*), true-positives (*orange*), and individuals with unknown SARS-CoV-2 status (*grey*) using the optimised cut-offs. The solid black line represents a linear regression for the positive measurements. (**a**) From left to right, association of EI-S1-IgG with the confirmatory test NT (*n*=354), GS-cPass (*n*=361), MG-RBD (*n*=272) and MG-N (*n*=355). We observed a population in the upper left quadrant, clearly negative in the confirmatory tests GS-cPass, MG-RBD and MG-N. (**b**) From left to right, association of Ro-N-Ig with the confirmatory test NT (*n*=362), GS-cPass (*n*=273), MG-RBD (*n*=354), and MG-N *n*=354).

To examine pre-test probability of assays following positive initial testing, the measurement values of all primary and confirmatory tests were correlated (Figs 6, S7–9). Overall, we observed high correlations, particularly for GS-cPass and MG-RBD with EI-S1-IgG, and MG-N with Ro-N-Ig (Fig. 6).

The categorical concordance for GS-cPass, MG-RBD, and MG-N with both Ro-N-Ig and EI-S1-IgG was similar (94 % or above), while the concordance of NT with both primary tests was lower (80%; Fig. 6). Concordances were improved by applying the optimised cut-offs, especially for VC-S1-IgG and VC-S2-IgG (Fig. S7).

## Discussion

We performed head-to-head comparisons of seven seroassays for SARS-CoV-2 and derived optimised cut-offs for several tests in a well-defined cohort with a total of 6658 samples [13, 14]. Although several reports have emerged that investigate the seroresponse to SARS-CoV-2 [4, 7, 17–25], only few feature direct head-to-head comparisons of many different assays in one set of samples [9, 11, 12, 26]. Studies have often used widely different patient populations, hampering direct comparisons of testing systems. In contrast, our study provides data generated from a representative cohort of the inhabitants of Munich, and not selected patient cohorts [13], allowing a more generalisable interpretation of results presented. When choosing the primary tests, we considered four main characteristics (i) availability in large quantities, (ii) enabled for at least semi-automated workup, (iii) acceptable pricing and iv) licenced for the use in europe. These criteria excluded tests such as VC-Array as they were too expensive (>20 EUR/test). GS-cPass was excluded, as at the time of the study it was not licensed for use yet, although this has changed by now, however still lacking automation. The EI-S1-IgG and IgA as well as Ro-N-Ig tests fulfilled all criteria and were thus applied.

Several studies have shown a relationship between disease severity and both antibody kinetics [6, 18, 20, 27, 28] and neutralisation capacity [17, 29–31]. Our data is derived from a population- and not primarily a patient based approach, thus the interpretation of the data is mainly for epidemiological use. Here, our data suggest that Ro-N-Ig performs more reliably than EI-S1-IgA and EI-S1-IgG, especially in low prevalence settings due to the lack of specificity in EI-S1-IgA and -IgG, similarly to previously published reports [4]. We would not recommend using serology to diagnose acute infections, as RT-PCR is positive during the acute phase of the infection and serology will become positive only later. Nevertheless, singling out subjects who require only one booster vaccination to save vaccination doses, or to identify possible re-infections might be important questions in direct patient care currently, besides epidemiological questions such as the assessment of possible herd immunity levels in the population.

In addition to the previously mentioned primary screening tests, we assessed confirmatory test performances using a subset of true-positive and true-negative samples, comparing assays targeting the highly-specific receptor binding domain (NT, GS-cPass, MG-RBD), which are considered direct or surrogate markers for viral neutralisation [32]. Our true-positive sample set was mainly derived from subjects with few to no symptoms, with often a rather low neutralising activity, allowing an in-depth cross-comparison of direct viral neutralisation (NT) with surrogate neutralisation markers (GS-cPass, MG-RBD) in oligo- or asymptomatic individuals. While NT is a direct representation of viral neutralisation, GS-cPass assesses the antibody-mediated inhibition of ACE2-interaction with SARS-CoV-2-S1-RBD and is therefore a cell-free surrogate neutralisation marker [33]. The cell-culture free tests performed particularly well with sensitivities of 96.3% for GS-cPass and 94.9% for MG-RBD, using the manufacturers’ thresholds. In contrast, NT performed sub-optimally with a sensitivity of 80.6%. A compelling explanation would be a rapid decline in neutralising capacity, which has been reported previously and is in line with our observations [30, 34]. As NT requires a complex BSL-3-laboratory infrastructure, it currently represents a critical bottleneck, while the surrogate tests can be performed under BSL2 conditions. Furthermore, NT might miss a substantial part of cases especially with lower titres, thus GS-cPass or MG-RBD might be considered in these cases as they offer similar specificity.

To investigate the potential yield from combining primary tests, we applied machine learning techniques (random forest and support vector machine, see Methods). However, these hardly improved the performance beyond what was achieved by Ro-N-Ig alone (Table 1). Similarly to previously published studies, we could not demonstrate an added value of performing more than one confirmatory test [4]. By extending our true positive cohort using combinations of two or more positive confirmatory tests, we repeated the analysis, using the raw value of the primary tests as a decision criterion. Some performance improvement could be achieved by combining EI-S1-IgG with GS-cPass or MG-RBD in the materials with raw values between 0.8 and 2.55 (22% of the samples). In these cases, overall accuracy improved from 93–98% and 99%, respectively. For further details on these combinations, see supplemental material (Table S5, Figs S10 and S11).

Situation-specific cut-off optimization has been proposed as a tool to improve seroassay performances for SARS-CoV-2 [4, 12]. We therefore derived optimised cut-offs based on the true-positive and true-negative cohorts; hereby, we were able to improve sensitivity in EI-S1-IgG and Ro-N-Ig, while specificity remained similar. Meyer *et al*. proposed optimised thresholds for EI-S1-IgG, and suggested evaluating the manufacturer’s cut-off before routine testing, highlighting the dilemma of securing both rule-in and rule-out properties to mitigate the risk of incorrect classification in a situation with highly-dynamic pre-test probabilities [4]. Whether these optimised cut-offs are generalisable remains uncertain: A seroprevalence study in Geneva compared both recommended and optimised cut-offs and did not observe any qualitative changes [25]. In our study, optimised cut-offs were similarly derived for confirmatory tests. Here, we could improve the sensitivity markedly for the VC-array with gains of >50% points for VC-N-IgG (39.8–94.4%) and close to 30% points for VC-S1-IgG (65.8–95.4%).

Even though some changes in performance estimates seem minimal, they might translate into a higher number of correctly classified diagnoses when testing is performed on a large scale. This is especially pertinent in low-prevalence settings, as particularly a high specificity is crucial to achieve a high positive predictive value. It may also be preferable to have a more sensitive cut-off for a primary test and confirm the positives with a highly-specific secondary test system [14].

In a systematic review by Huang *et al*. in 2020, the median detection time across different antibodies against SARS-CoV-2 was 11 days, similar to SARS-CoV-1 [6]. We therefore additionally assessed the seropositivity stratified with the date of the first positive RT-PCR test. In our cohort of mostly oligosymptomatic true-positive subjects, 11.4% (22/193) were not detected in the primary serological tests, with a third of those being <30 days after positive RT-PCR. Overall, in our dataset of samples >30 days after positive RT-PCR, a modest 8.1% (13/160) remained negative. Late or lacking seroconversion has been described previously, mostly in oligo- or asymptomatic subjects [31, 35], and authors have speculated about vastly varying proportions of subjects unable to mount an antibody response detectable by commonly used assays [20, 21, 36, 37].

Different studies have reported a rapid decline of antibody titres over time [20, 30, 34]. In our cohort, antibody levels measured with EI-S1-IgA declined early on, most pronouncedly within the first 30 days, resulting in 40% of the subjects being below the positive cut-off >30 days after RT-PCR positivity. In contrast, antibody levels detected with EI-S1-IgG remained stable and detectable over longer periods. Moreover, titres measured with Ro-N-Ig increased, with more than 80% of true-positive subjects being rated as positive >90 days. Studies published so far show ambiguous results, partly suggesting that overall, antibody responses to S might be more stable than responses to N [38]; our results suggest that the observed differences are more attributed to the testing approach than the antigen itself.

Our study has several limitations. The sample set is derived from a representative cohort in Munich, Germany. Despite being an ethnically diverse city, the results presented here might not be representative of other geographical regions. Additionally, it was not feasible to perform all confirmatory tests on all samples; a subset, namely those with positive results in at least one primary test as well as a known negative/positive cohort, were tested using these systems. Finally, we did not have information on underlying health conditions of all subjects, e.g. conditions known to affect the quantity of polyclonal antibodies.

In conclusion, our study provides a cross-comparison of seven different widely used serological assays for SARS-CoV-2 and proposes new cut-offs for several tests. This study can be used as a resource to enable the refinement of testing strategies for individual and public-health use. Our approach presented here used a well-defined sample set with true-positive as well as true-negative specimens. Subsequently, we extrapolated the established findings to samples derived from a population-based seroprevalence cohort and were therefore able to generate a robust head-to-head comparison for diagnostic performance estimates of several serological tests for SARS-CoV-2.

## Supplementary Data

Supplementary material 1
