# Supplementary material for: Head-to-head evaluation of seven different seroassays including direct viral neutralisation in a representative cohort for SARS-CoV-2
Source: J Gen Virol. 2021 Oct 8;102(10):001653. doi: 10.1099/jgv.0.001653 (PMC8604188; doi:10.1099/jgv.0.001653)
Supplement: Supplementary material 1 [file jgv-102-1653-s001.pdf]

Appendix and Supplementary Material:

Cohorts

Samples for testing were derived from three different sample sets (supplemental table 1, figure 1). For known negatives (true-negatives), specimens from 991 healthy blood donors, sampled at two distinguished time points were used (i. October 2019, to represent the pre-common cold period and ii. March 2020, pre-COVID19 and post-common cold season). For known positives (true-positives), volunteers ≥18 years that tested RT-PCR-positive for SARS-CoV-2 in Munich were sampled (n=193). Furthermore, specimens from the Prospective COVID-19 Cohort Munich (KoCo19) were included in the analyses (n=5,474; of which an additional subset of 100 true-negatives was identified). In total, 6,658 samples were included in the analyses for the primary tests (Euroimmun Anti-S1-SARS-CoV-2-ELISA-IgA (EI-S1-IgA; n=6,657), Euroimmun Anti-SARS-CoV-2-ELISA-IgG (EI-S1-IgG; n=6,658), and Elecsys Anti-SARS-CoV-2 Roche N pan−Ig (Ro-N-Ig; n=6,636)) (supplemental table 2). A large part of samples that tested positive in primary tests, in addition to a subset of true-negatives, were subjected to confirmatory testing (n=362; figure 1, supplemental table 2).

Assay Performances

Euroimmun anti-S1 IgA/IgG (Euroimmun Anti-SARS-CoV-2-ELISA):

EI-S1-IgG and EI-S1-IgA test kits were utilised according to the manufacturer's instructions using an Euroanalyser-1 robot (Euroimmun, Lübeck, Germany). Raw values presented in all figures of the manuscript are the quotients of the optical density measurements given by the manufacturer’s software.

Roche anti-N pan-Ig (Elecsys Anti-SARS-CoV-2):

Ro-N-Ig (Roche, Mannheim, Germany) testing was conducted in accordance with the manufacturer’s guidelines. Measurements were performed on the cobas 400-e411 and/or 8000-e801 modules (Roche, Basel, Switzerland) there were no differing classifications between two employed instruments (Figure 2B, Supplemental Figure 1B) and the performance of both instruments was comparable. Values indicated in all tests correspond to the Cut-Off-Index (COI) of the individual samples.

GenScript® cPass:

SARS-CoV-2 surrogate virus neutralisation test (GenScript®, Piscataway, New Jersey, USA; hereafter called ”GS-cPass”) was performed according to the manufacturer’s instructions. Photometric measurements were performed using the Tecan Sunrise (Tecan, Männedorf, Switzerland). Binding inhibition was calculated in percentage.

Micro neutralisation (NT):

Manual micro virus neutralisation assay (NT) analysis was performed in 96-well culture plates (Greiner bio-one, Frickenhausen, Germany) as previously described, with the exception that confluent cells were incubated instead of adding cells after the neutralisation reaction.[1] Samples with a titre < 1:5 dilution were classified as "NT negative" and samples with a titre ≥1:5 were classified as "NT positive". In brief, virus stocks (50 TCID/50 µl) were prepared from SARS-CoV-2 cultured samples (1st passage, strain MUC IMB-1, cultured in Vero E6 cells) and stored at -80° C for later use. Plasma samples were diluted twofold from of 1:5 to 1:80 in Minimal Essential Medium (MEM, plus Non-Essential Amino Acids Solution and Antibiotic-Antimycotic Solution; all Invitrogen, Thermo Fisher Scientific, Darmstadt, Germany) and tested in duplicates. On each plate, a known positive and a known negative plasma sample were tested as controls. In addition, a mock control and a virus back-titration were added to each plate. The subsequently diluted plasma samples were pre-incubated with virus for one hour at 37°C in 5% CO_2 and 95% humidity. The suspension was then transferred into the wells with the confluent Vero-E6 cells. After 72 hours of incubation at 37°C (5% CO_2), the supernatants was discarded and the plates were fixed and stained in a solution of 13% formalin/PBS plus 0.1% crystal violet. The highest plasma dilution showing complete inhibition of CPE in both microtitre plate wells corresponded to the NAbs titre.

VIRAMED SARS-CoV-2 ViraChip® microarray:

SARS-CoV-2 ViraChip® microarray (VIRAMED Biotech AG, Planegg, Germany; hereafter called VC-N-IgA/IgM/IgG; VC-S1-IgA/IgM/IgG; VC-S2-IgA/IgM/IgG) was based on an enzyme-immunoassay for the qualitative detection of IgG, IgA or IgM antibodies against the specific SARS-CoV-2 recombinant antigens S1, S2, and N in human serum. The assay was performed on a Dynex ELISA Processor DSX® (Dynex Technologies, Denkendorf, Germany) according to the manufacturer's instructions. We have reported the values provided by the automated ELISA processor, which are in arbitrary units.

Mikrogen *recom*Line SARS-CoV-2 IgG line immunoassay:

The *recom*Line SARS-CoV-2 IgG line immunoassay (Mikrogen, Neuried, Germany; hereafter named MG-S1, MG-N, MG-RBD) was based on nitrocellulose strips with recombinant SARS-CoV-2 antigens S1, N and RBD as well as recombinant N antigens derived from seasonal human CoV NL63, OC43, 229E, and HKU1. The assay for the qualitative detection of human IgG against the respective antigens was performed using the fully automated *recom*Line strip processor Carl (Mikrogen, Neuried, Germany) according to the manufacturer's instructions. The raw values presented are in arbitrary units. The test results below the cut-off of 1 are categorised as negative and information regarding their quantitative values is comitted.

Statistical Analysis

Optimisation of cut-off values

Using the raw values of both the primary and confirmatory tests from the true-positive and true-negative cohorts, several statistical techniques were applied to identify optimised cut-offs thresholds and calculate the performance of classifiers based on single tests or combinations of tests.

The EI-S1-IgA, EI-S1-IgG, and Ro-N−Ig tests were available for 1,266 subjects with known SARS-CoV-2 status (table 1 and supplemental table 2). For better comparability, 18 samples that had no available result for Ro-N-Ig were omitted. As these tests were also conducted on all KoCo19 study subjects, the results for classifiers based on these tests could be used to predict the serological status of the Munich cohort and to adjust the crude seroprevalence estimates based on the specificity and sensitivity of these classifiers.[2] The sample sizes available for the confirmatory tests are given in table 1 and supplemental table 2.

For each of the single tests, a nonparametric bootstrap approach was used to optimise the cut-off threshold as well as estimate the corresponding specificity, sensitivity and overall prediction accuracy. More precisely, 10,000 bootstrap samples were drawn (with replacement, without balancing positive/negative true test outcomes). For each, a cut-off was determined that maximised the overall prediction accuracy. We refer to cut-offs obtained in single bootstrap samples as bootstrap cut-offs to distinguish them from the final optimised cut-off. The latter is eventually obtained by taking the median of 10,000 bootstrap cut-offs.

Some of the tests yield left-censored measurements, i.e., values below a detection limit are not resolved but summarised in one category. To determine each bootstrap cut-off, we replaced the left-censored values in each bootstrap test with a random realisation using a uniform distribution between zero and the detection limit. The optimisation with the resulting virtual uncensored sample can yield bootstrap cut-offs above the detection limit or below. While the former values were accepted, the latter underwent post-processing as they cannot be implemented in serological studies. For bootstrap cut-offs below the detection limit we evaluated the classification accuracy for the interval bounds – i.e., zero and detection limit – which could be implemented. The bounds yielding a higher prediction accuracy was used as bootstrap cut-off for the respective bootstrap sample.

For each test, the bootstrap cut-off was used to predict the serological status of the observations not included in the respective bootstrap sample (out-of-bag observations). After considering the predictions for each observation over all bootstrap replications, majority votes were chosen as final single predictions. These predictions were compared to the true serological status to calculate specificity, sensitivity and overall prediction accuracy. To this end, the optimised cut-off and the three performance criteria were estimated based on different data, namely, the former was estimated based on bootstrap samples and the latter were estimated based on the respective out-of-bag observations.

The performance of the optimised cut-off threshold was compared to the performance of the cut-off determined by the manufacturer. Since the manufacturer calibrated the cut-off using their own data, no bootstrap was applied to this: The three performance indices were computed by comparing the true serological status to the predictions based on the cut-off for the entire sample. Overall, the calculation of the performance indices for both optimised and manufacturer's cut-offs rely on out-of-sample predictions. The difference in the validation data sets may lead to differences in performance indices even if both the optimised and the manufacturer's cut-offs are the same. We know that while the term "optimised cut-offs" suggest that they are better, it can be that the manufacturer cut-offs reach bye chancge a higher sensitivity, specificity or overall accuracy on the dataset. This can happen as the optimisation is performed on the bootstrapped data, is however only the case for situations in which both cut-offs anyhow perform very similar.

To evaluate the potential benefit of combining several primary tests, we trained a random forest and a support vector machine which took the measurements for all three primary tests (EI-S1-IgA, EI-S1-IgG, and Ro-N−Ig) as inputs. In order to tuneing the parameters for the random forest, the number of selected variables at each split and the minimum node size were taken into account. For the support vector machine, a Gaussian radial basis function kernel along with the kernel coefficient gamma and the penalty parameter of the error term as tuning parameters were selected. For both the random forest and the support vector machine, a grid search was applied to find the combination of the two tuning parameters that minimised the out-of-bag prediction error resulting from 1,000 bootstrap samples. The final estimates for the performance criteria were then obtained by running the respective classifier with the optimised tuning parameters using 2,000 bootstrap replicates and the majority votes from the resulting out-of-bag predictions, as described above regarding the classifiers based on a single test.

Software

For analyses and visualisations, we used the R software, version 4.0.2. For data visualisation, we used in particular the packages "ggplot2"[3] and "RainCloudPlots"[4].

References

1. Haselmann V, Özçürümez MK, Klawonn F, et al. Results of the first pilot external quality assessment (EQA) scheme for anti-SARS-CoV2-antibody testing. *Clinical chemistry and laboratory medicine* 2020.
2. Pritsch M, et al. Prevalence and risk factors of infection in the representative COVID-19 cohort Munich. *Adjacent manuscript*.
3. Wickham H. ggplot2: elegant graphics for data analysis: springer; 2016.
4. Allen M, Poggiali D, Whitaker K, Marshall T, Kievit R. Raincloud plots: a multi-platform tool for robust data visualization. Wellcome Open Res. 4, 63. 2019.

Nature of cohort	Recruitment and study details	Sample numbers and definitions	Days from positive PCR median (min-max; mean)
Healthy blood donors (=known SARS-CoV-2 negatives)	October 2019	Total n=500<br>*True-positives = 0*<br>*True-negatives = 500*<br>*Unknown = 0*	NA
	March 2020 *(after common cold season)*	Total n=491<br>*True-positives = 0*<br>*True-negatives = 491*<br>*Unknown = 0*	NA
SARS-CoV-2 infected subjects (=known SARS-CoV-2 positives)	Volunteers >18 yrs that tested positive for SARS-CoV-2 in Munich (156 households)	Total n=193<br>*True-positives = 193*<br>*True-negatives = 0*<br>*Unknown = 0*	n=187<br><br>median 99 days (7-126; mean = 86)
Representative population-based study & substudies (Prospective COVID-19 Cohort Munich; KoCo19),	3,003 randomly selected Munich households (April-June 2020) & 92 households from substudies	Total n=5474<br>*True-positives = 0*<br>*True-negatives = 100*<br>*Unknown = 5374*	NA
**Total samples used**	**Total n = 6658**	***True-positives = 193***<br>***True-negatives = 1091***<br>***Unknown = 5374***	

***Supplemental Table 1*: Cohort details.**
Characterisation of study participants. Subjects with a positive RT-PCR were considered as true-positives, while blood donors (sampled in the pre-COVID-19 era) were classified as true-negatives. In addition, we included subjects recruited into the Representative COVID-19 Cohort Munich (KoCo19), 100 of which were considered as true-negatives. For subjects with several longitudinal measurements, the blood sample with the most complete dataset was retained. For similar datasets, the earliest measurement was considered. For operational replicates, the latest measurement was used.

Test	Sample size	Positive result optim. / manuf. cut-off	Negative result optim. / manuf. cut-off	True-positives	True-negatives
EI-S1-IgA	6,657	687 / 674	5970 / 5983	193	1091
EI-S1-IgG	6,658	321 / 309	6337 / 6349	193	1091
RO-N-Ig	6,636	307 / 289	6329 / 6347	193	1073
NT	355	166*	189	108	106
GS-cPass	355	197 / 197	158 / 158	108	106
VC-N-IgA	361	NA / 26	NA / 355	108	110
VC-N-IgM	362	NA / 18	NA / 344	108	111
VC-N-IgG	362	125 / 81	237 / 281	108	111
VC-S1-IgA	361	NA / 37	NA / 324	108	110
VC-S1-IgM	362	NA / 17	NA / 345	108	111
VC-S1-IgG	362	201 / 134	161 / 228	108	111
VC-S2-IgA	361	NA / 29	NA / 332	108	110
VC-S2-IgM	362	NA / 15	NA / 347	108	111
VC-S2-IgG	362	145 / 37	217 / 225	108	111
MG-NP	273	139 / 139	134 / 134	78	106
MG-RBD	273	137 / 137	136 / 136	78	106
MG-S1	273	141 / 141	132 / 132	78	106
229E	273	NA / 129	NA / 144	78	106
NL63	273	NA / 127	NA / 146	78	106
OC43	273	NA / 79	NA / 194	78	106
HKU1	273	NA / 130	NA / 143	78	106

***Supplemental Table 2*: Number and description of tests.**

For subjects with multiple blood samples and test results from different time points, measurements were excluded (only one value per individual and test system). Confirmatory tests were performed on a subset of samples. Samples from true-positives and true-negatives were used as controls for confirmatory tests.

* For NT, dilutions starting at 1:5 were used (see Methods).

Assay	Instrument(s) used	Readout
EI-S1-IgG	Euroanalyser-1 (Euroimmun, Lübeck, Germany)	quotient of the optical density
EI-S1-IgA	Euroanalyser-1 (Euroimmun, Lübeck, Germany)	quotient of the optical density
Ro-N-Ig	cobas 400-e411 (Roche, Basel, Switzerland)	cut-off-index (COI)
	cobas 8000-e801 (Roche, Basel, Switzerland)	cut-off-index (COI)
NT	NA	dilution
GS-cPass	Tecan Sunrise (Tecan, Männedorf, Switzerland)	inhibition [%]
VC-N-IgA/IgM/IgG	Dynex ELISA Processor DSX® (Dynex Technologies, Denkendorf, Germany)	arbitrary unit
VC-S1-IgA/IgM/IgG	Dynex ELISA Processor DSX® (Dynex Technologies, Denkendorf, Germany)	arbitrary unit
VC-S2-IgA/IgM/IgG	Dynex ELISA Processor DSX® (Dynex Technologies, Denkendorf, Germany)	arbitrary unit
MG-S1	recomLine strip processor Carl (Mikrogen, Neuried, Germany)	quotient and category (<1)
MG-N	recomLine strip processor Carl (Mikrogen, Neuried, Germany)	quotient and category (<1)
MG-RBD	recomLine strip processor Carl (Mikrogen, Neuried, Germany)	quotient and category (<1)

***Supplemental Table 3*: Platform used and units given for the respective assays**

Group comparison	Adjusted p value		
	El-S1-IgA	El-S1-IgG	Ro-N-Ig
Up to 30 days - Between 30-90 days	0.015*	1.000	0.023*
Up to 30 days - After 90 days	0.025*	1.000	0.001***
Between 30-90 days - After 90 days	1.000	1.000	0.619

***Supplemental Table 4*: Pairwise differences referred to Figure 3**

Combination:	True positives (n)	True negatives (n)	Sensitivity primary only	Specificity primary only	Accuracy primary only	Sensitivity test combination	Specificity test combination	Accuracy test combination	Fraction confirmatory tests performed	Lower bound on primary test for confirmatory test	Upper bound on primary test for confirmatory test
EI-S1-IgG & GS-cPass	138	130	0.99	0.87	0.93	0.98	0.98	0.98	0.22	0.8	2.55
Ro-N-Ig & GS-cPass	138	130	0.99	0.97	0.98	1.00	1.00	1.00	0.08	0.08	2.09
EI-S1-IgG & MG-RBD	137	130	0.99	0.86	0.93	0.99	0.98	0.99	0.22	0.8	2.55
Ro-N-Ig & MG-RBD	137	130	0.99	0.97	0.98	1.00	1.00	1.00	0.08	0.08	2.09
EI-S1-IgG & MG-N	137	130	0.99	0.86	0.93	0.98	0.98	0.98	0.21	0.8	2.55
Ro-N-Ig & MG-N	137	130	0.99	0.96	0.98	0.97	0.99	0.98	0.04	0.38	2.09

Supplemental Table 5**:** Possible combinations between primary tests EI-S1-IgG and Ro-N-Ig respectively with the confirmatory tests GS-cPass, MG-RBD and MG-N.

Assay	Confirmatory tests performed			Negatives according to confirmatory test		
	EI-S1-IgA	*EI-S1-IgG*	*Ro-N-Ig*	*EI-S1-IgA*	*EI-S1-IgG*	*Ro-N-Ig*
LineBlot_NP_SARS_2	1	0	2	1	0	2
LineBlot_RBD_SARS_2	1	0	2	1	0	2
LineBlot_S1_SARS_2	1	0	2	1	0	2
VC_S1_IgA	3	2	2	3	2	2
VC_S1_IgG	3	2	2	3	2	2
VC_S2_IgA	3	2	2	2	2	1
VC_S2_IgG	3	2	2	3	2	2
VC_N_IgA	3	2	2	2	2	0
VC_N_IgG	3	2	2	3	1	0
cPass	1	0	2	1	0	2
NT	1	0	2	1	0	2

***Supplemental Table 6*: Confirmatory tests (rows) performed for true-negatives that are positive according to primary test (columns)**

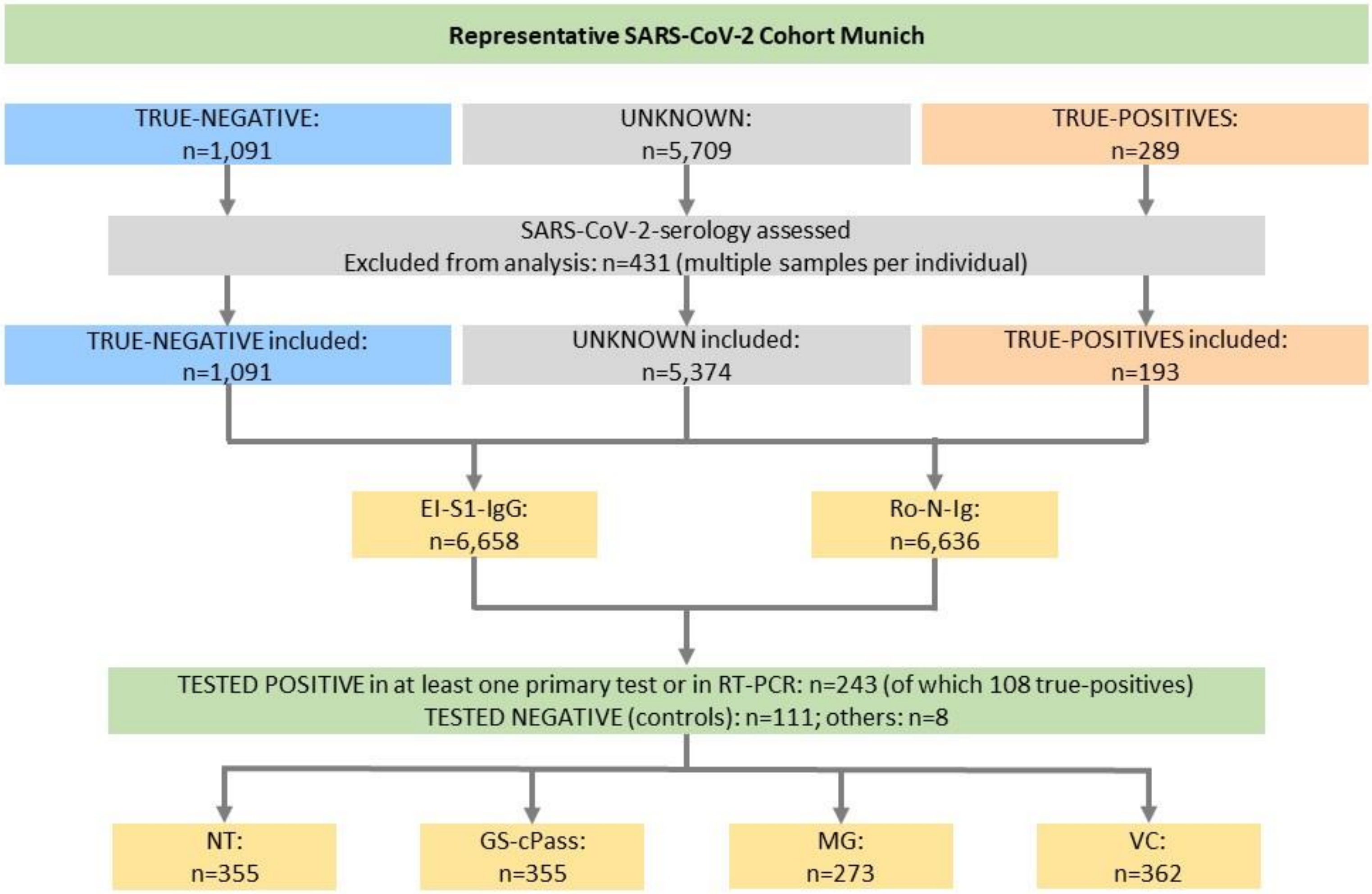

Supplemental Figure 1: Cohort composition and characterisation of the study participants.
True-positives were defined as subjects with a positive RT-PCR; true-negatives as blood donors from the pre-COVID-19 era. In addition, we included individuals recruited into the Representative COVID-19 Cohort Munich (KoCo19), 100 of which were considered as true-negatives. For each participant, a single sample was used for statistical analysis. Among individuals with longitudinal measurements, the blood sample with the most complete dataset was retained. For similar datasets, the earliest measurement was considered. For operational replicates, the latest measurement was used.

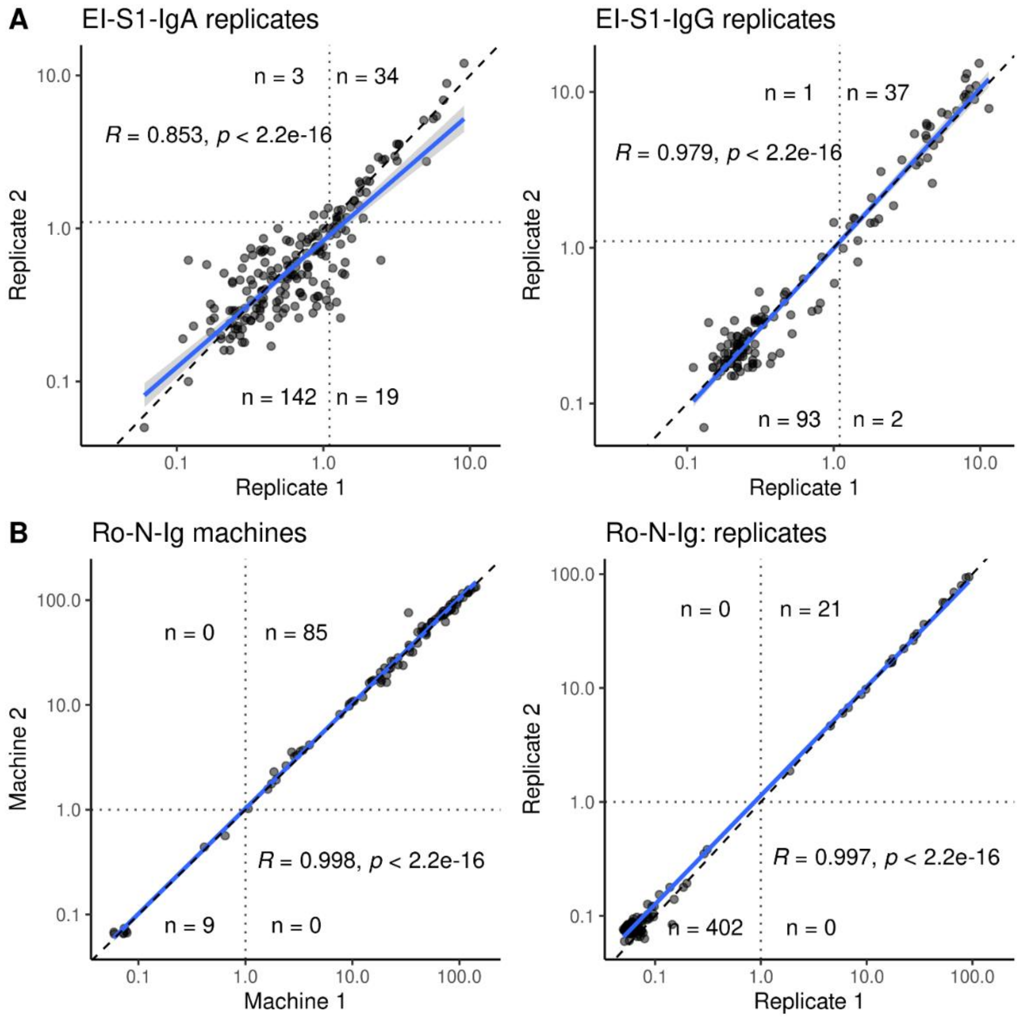

***Supplemental Figure 2*: Reliability analysis of operational replicates.**
Discrepant/non-discrepant results derived from the same blood samples measured in two replicates. Re-measuring the samples confirmed a correlation of R=1 for the quantitative measurement values without a single classification change (0/423).
(A) Scatterplot of replicates of EI-S1-IgA (left) and EI-S1-IgG(right). The correlation for EI-S1-IgA was R=0.853, but classification was discrepant in 22 cases (11.1%). The correlation for EI-S1-IgG was higher with R=0.979, with 3 subjects changing category (2.3%). Replicates for both tests were performed on the same platform; however, with varying lot numbers and operators. The changes from positive to negative status in EI-S1-IgA was most likely caused by a batch effect.
(B) Scatterplot of replicates of Ro-N-Ig between modules e411 (machine 1) and e801 (machine 2) (left), and on module e801 (right). Although the 511 samples were tested on two different modules with kits of varying lot numbers and operators, the correlation was R=0.99, with not a single discrepant classification.

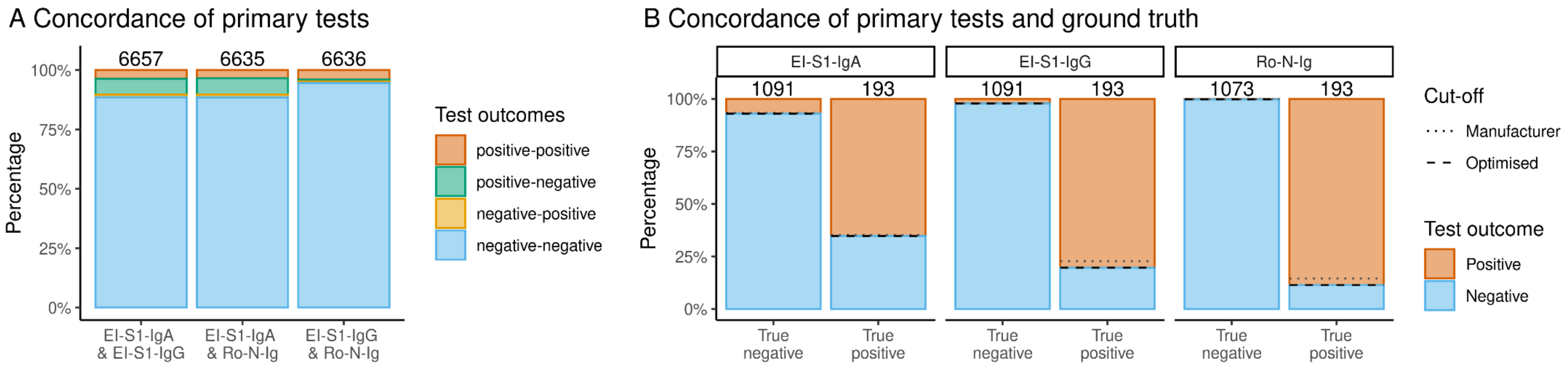

Supplemental Figure 3: Concordance of primary tests.
(A) Concordance of primary tests based on manufacturer's cut-offs. Numbers of paired samples are indicated above the bars. Unspecific reactivities are represented in green and yellow.
(B) Test results of the primary tests based on optimised cut-offs and ground truth.

Suppl.Fig.4A

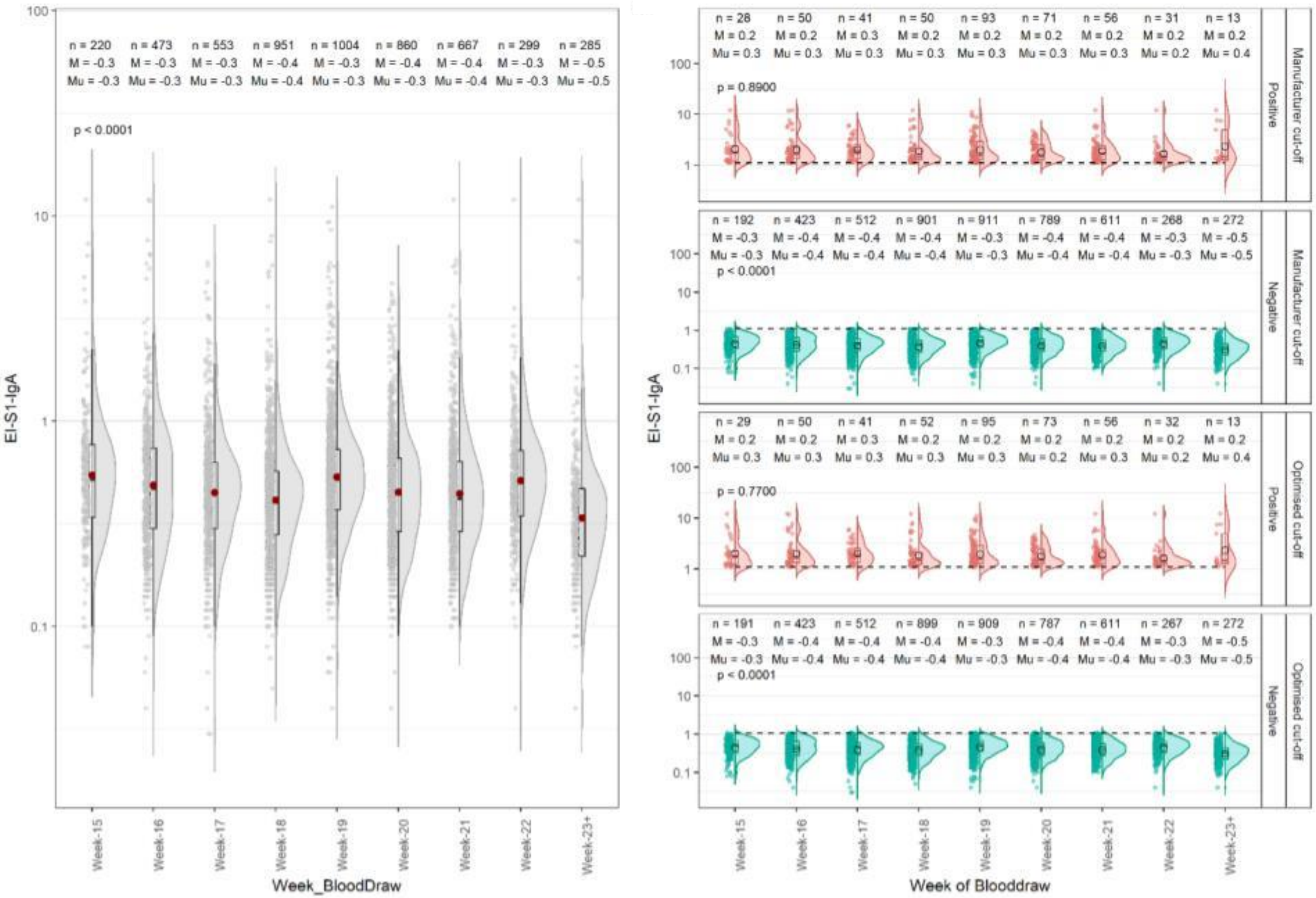
n = 220 n = 473 n = 553 n = 951 n = 1004 n = 860 n = 667 n = 299 n = 285
M = -0.3 M = -0.3 M = -0.3 M = -0.4 M = -0.3 M = -0.4 M = -0.4 M = -0.3 M = -0.5
Mu = -0.3 Mu = -0.3 Mu = -0.3 Mu = -0.4 Mu = -0.3 Mu = -0.3 Mu = -0.4 Mu = -0.3 Mu = -0.5
p < 0.0001
100
10
1
0.1
El-S1-IgA
Week-15 Week-16 Week-17 Week-18 Week-19 Week-20 Week-21 Week-22 Week-23+
Week_BloodDraw
n = 28 n = 50 n = 41 n = 50 n = 93 n = 71 n = 56 n = 31 n = 13
M = 0.2 M = 0.2 M = 0.3 M = 0.2 M = 0.2 M = 0.2 M = 0.2 M = 0.2 M = 0.2
Mu = 0.3 Mu = 0.3 Mu = 0.3 Mu = 0.3 Mu = 0.3 Mu = 0.3 Mu = 0.3 Mu = 0.2 Mu = 0.4
p = 0.8900
Positive
Manufacturer cut-off
n = 192 n = 423 n = 512 n = 901 n = 911 n = 789 n = 611 n = 268 n = 272
M = -0.3 M = -0.4 M = -0.4 M = -0.4 M = -0.3 M = -0.4 M = -0.4 M = -0.3 M = -0.5
Mu = -0.3 Mu = -0.4 Mu = -0.4 Mu = -0.4 Mu = -0.3 Mu = -0.4 Mu = -0.4 Mu = -0.3 Mu = -0.5
p < 0.0001
Negative
Manufacturer cut-off
n = 29 n = 50 n = 41 n = 52 n = 95 n = 73 n = 56 n = 32 n = 13
M = 0.2 M = 0.2 M = 0.3 M = 0.2 M = 0.2 M = 0.2 M = 0.2 M = 0.2 M = 0.2
Mu = 0.3 Mu = 0.3 Mu = 0.3 Mu = 0.3 Mu = 0.3 Mu = 0.2 Mu = 0.3 Mu = 0.2 Mu = 0.4
p = 0.7700
Positive
Optimised cut-off
n = 191 n = 423 n = 512 n = 899 n = 909 n = 787 n = 611 n = 267 n = 272
M = -0.3 M = -0.4 M = -0.4 M = -0.4 M = -0.3 M = -0.4 M = -0.4 M = -0.3 M = -0.5
Mu = -0.3 Mu = -0.4 Mu = -0.4 Mu = -0.4 Mu = -0.3 Mu = -0.4 Mu = -0.4 Mu = -0.3 Mu = -0.5
p < 0.0001
Negative
Optimised cut-off
El-S1-IgA
Week-15 Week-16 Week-17 Week-18 Week-19 Week-20 Week-21 Week-22 Week-23+
Week of Blooddraw

Suppl.Fig.4B

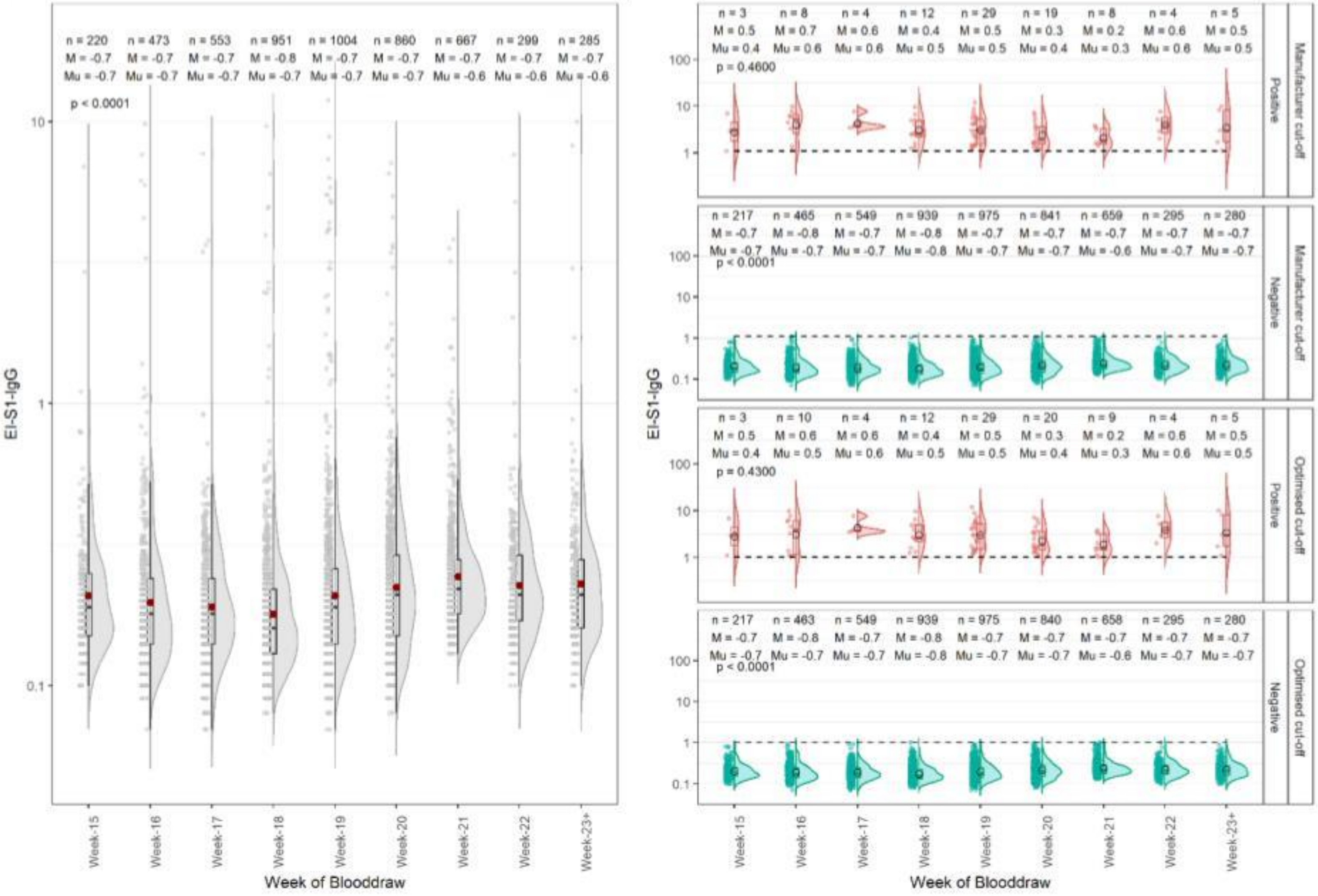
n = 220 M = -0.7 Mu = -0.7
n = 473 M = -0.7 Mu = -0.7
n = 553 M = -0.7 Mu = -0.7
n = 951 M = -0.8 Mu = -0.7
n = 1004 M = -0.7 Mu = -0.7
n = 860 M = -0.7 Mu = -0.7
n = 667 M = -0.7 Mu = -0.6
n = 299 M = -0.7 Mu = -0.6
n = 285 M = -0.7 Mu = -0.6
p < 0.0001
EI-S1-IgG
Week of Blooddraw
Week-15
Week-16
Week-17
Week-18
Week-19
Week-20
Week-21
Week-22
Week-23+
Manufacturer cut-off
Optimised cut-off
Positive
Negative
p = 0.4600
p = 0.4300
p < 0.0001

Suppl.Fig.4C

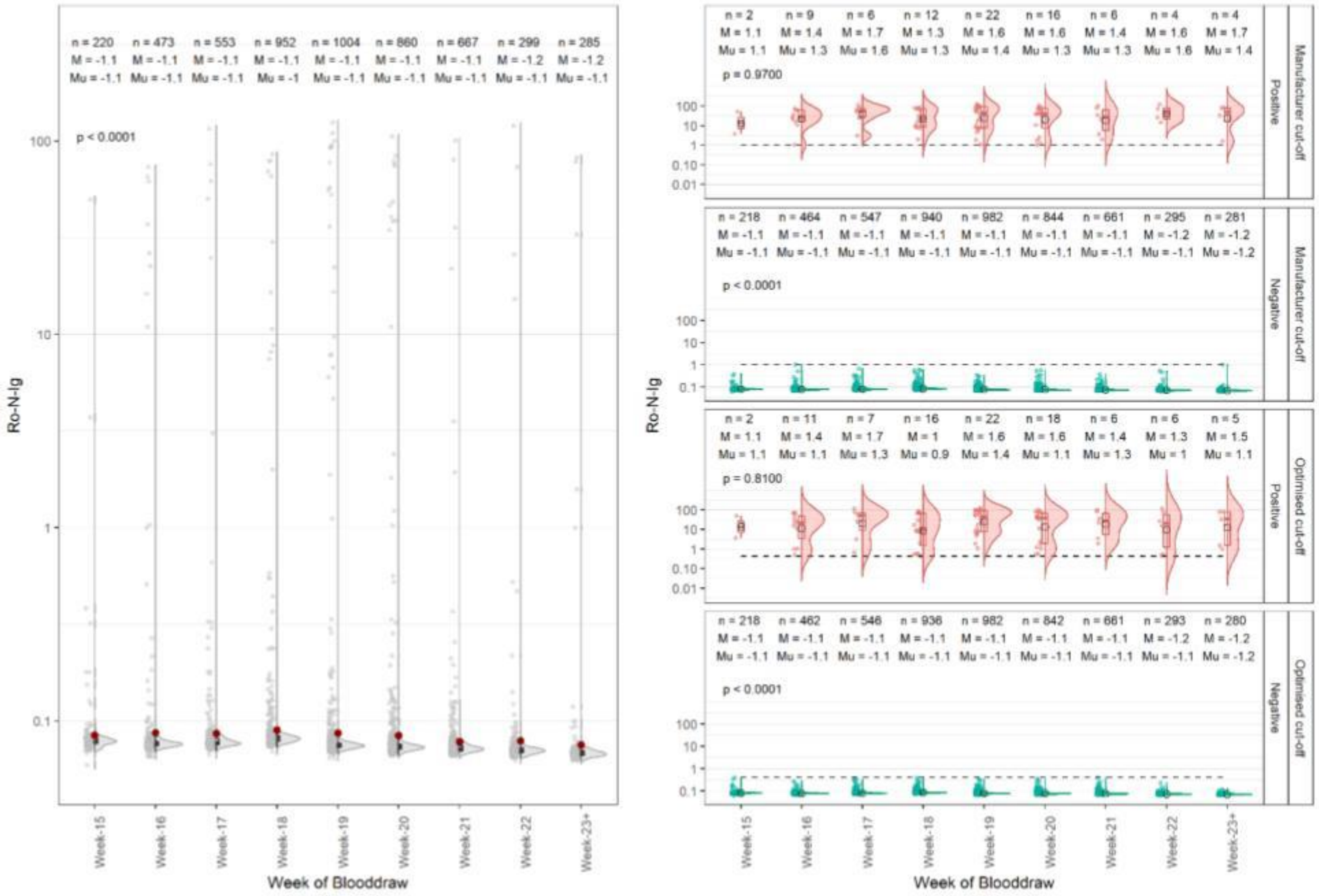
n = 220 n = 473 n = 553 n = 952 n = 1004 n = 860 n = 667 n = 299 n = 285
M = -1.1 M = -1.1 M = -1.1 M = -1.1 M = -1.1 M = -1.1 M = -1.1 M = -1.2 M = -1.2
Mu = -1.1 Mu = -1.1 Mu = -1.1 Mu = -1 Mu = -1.1 Mu = -1.1 Mu = -1.1 Mu = -1.1 Mu = -1.1
p < 0.0001
Ro-N-Ig
Week of Blooddraw
Week-15 Week-16 Week-17 Week-18 Week-19 Week-20 Week-21 Week-22 Week-23+
Manufacturer cut-off
Optimised cut-off
Positive
Negative
n = 2 n = 9 n = 6 n = 12 n = 22 n = 16 n = 6 n = 4 n = 4
M = 1.1 M = 1.4 M = 1.7 M = 1.3 M = 1.6 M = 1.6 M = 1.4 M = 1.6 M = 1.7
Mu = 1.1 Mu = 1.3 Mu = 1.6 Mu = 1.3 Mu = 1.4 Mu = 1.3 Mu = 1.3 Mu = 1.6 Mu = 1.4
p = 0.9700
n = 218 n = 464 n = 547 n = 940 n = 982 n = 844 n = 661 n = 295 n = 281
M = -1.1 M = -1.1 M = -1.1 M = -1.1 M = -1.1 M = -1.1 M = -1.1 M = -1.2 M = -1.2
Mu = -1.1 Mu = -1.1 Mu = -1.1 Mu = -1.1 Mu = -1.1 Mu = -1.1 Mu = -1.1 Mu = -1.1 Mu = -1.2
p < 0.0001
n = 2 n = 11 n = 7 n = 16 n = 22 n = 18 n = 6 n = 6 n = 5
M = 1.1 M = 1.4 M = 1.7 M = 1 M = 1.6 M = 1.6 M = 1.4 M = 1.3 M = 1.5
Mu = 1.1 Mu = 1.1 Mu = 1.3 Mu = 0.9 Mu = 1.4 Mu = 1.1 Mu = 1.3 Mu = 1 Mu = 1.1
p = 0.8100
n = 218 n = 462 n = 546 n = 936 n = 982 n = 842 n = 661 n = 293 n = 280
M = -1.1 M = -1.1 M = -1.1 M = -1.1 M = -1.1 M = -1.1 M = -1.1 M = -1.2 M = -1.2
Mu = -1.1 Mu = -1.1 Mu = -1.1 Mu = -1.1 Mu = -1.1 Mu = -1.1 Mu = -1.1 Mu = -1.1 Mu = -1.2
p < 0.0001

Supplemental Figure 4**: Temporal analysis by recruitment week.**

The time-dependent distribution of the read-out values for (A) EI-S1-IgA, (B) EI-S1-IgG.and (C) Ro-N-Ig. The distribution of all raw values (in grey on the left side), as well as an individual analysis of positively (red) and negatively (green) tested samples was evaluated. Distributions are depicted for the manufacturer's cut-off (first two figures, upper right) as well as the optimised cut-off (last two figures, lower right). n denotes the count of the outcomes, M the median (also observed as the horizontal line in the boxplot) and Mu the mean (denoted by the red dot) for each calendar week in the study. Our analyses confirm that within our sample set, the measured background does not vary considerably within the prospective sample period of the KoCo19 study.

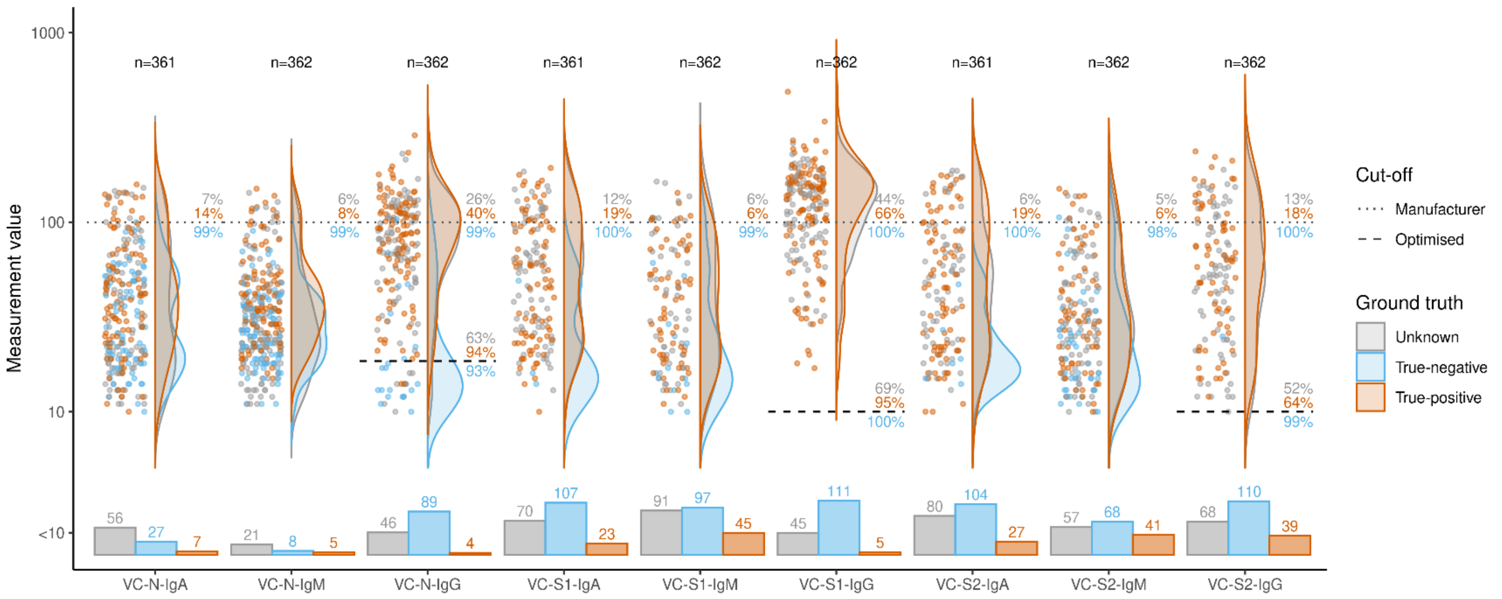

Supplemental Figure 5: Distribution VC.

Distributions of the test results for true-negatives (blue), true-positives (orange), and individuals with unknown SARS-CoV-2 status (grey). Black dotted and dashed lines represent the original manufacturer's and the optimised positivity cut-offs. Orange (blue) numbers give the percentage of true-positives (true-negatives) correctly detected by the test. The orange values above the dotted line represents the percentage of positive test results for the true positive cohort, the blue number below demonstrate the percentages of negatives in the true negative cohort. Grey numbers indicate the percentages of positive samples with unknown SARS-CovV-2. These percentages were calculated over the total number of samples with unknown SARS-CoV-2 with available test results. Bar charts below violin plots represent the information for the categorical part of the test.

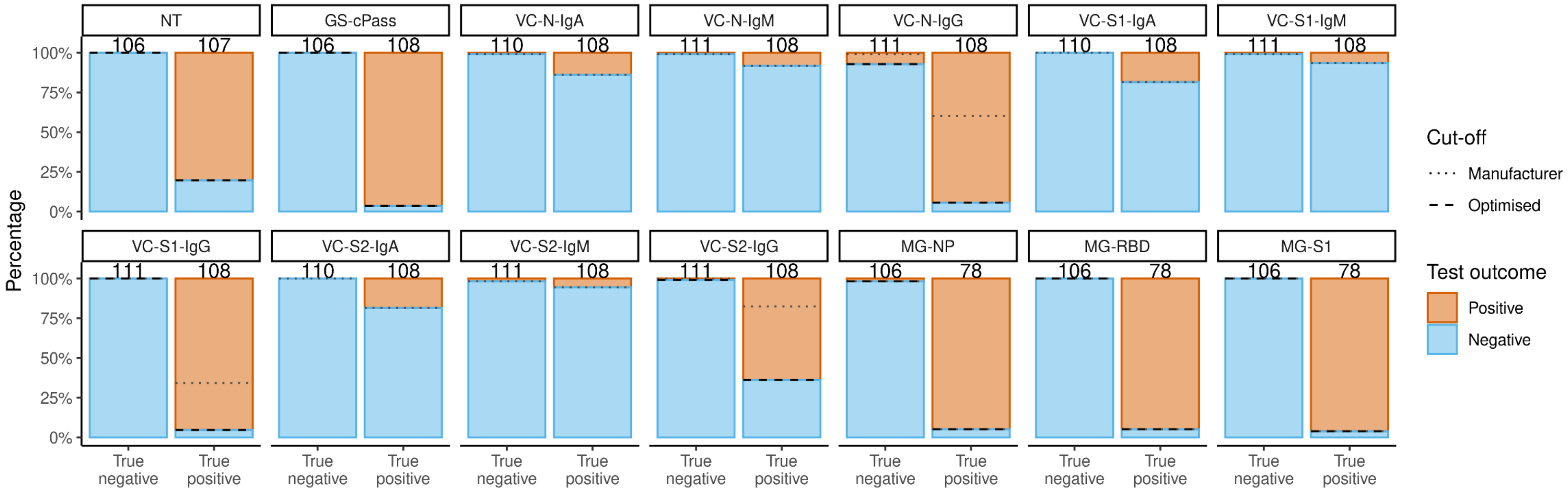

Supplemental Figure 6: Description and comparison of performances of confirmatory tests.
Test results according to ground truth colour coded by optimised cut-off for the primary tests. The dotted lines represent the manufacturer's cut-offs, the dashed lines the optimised cut-offs defined within this study.

Suppl.Fig.7A

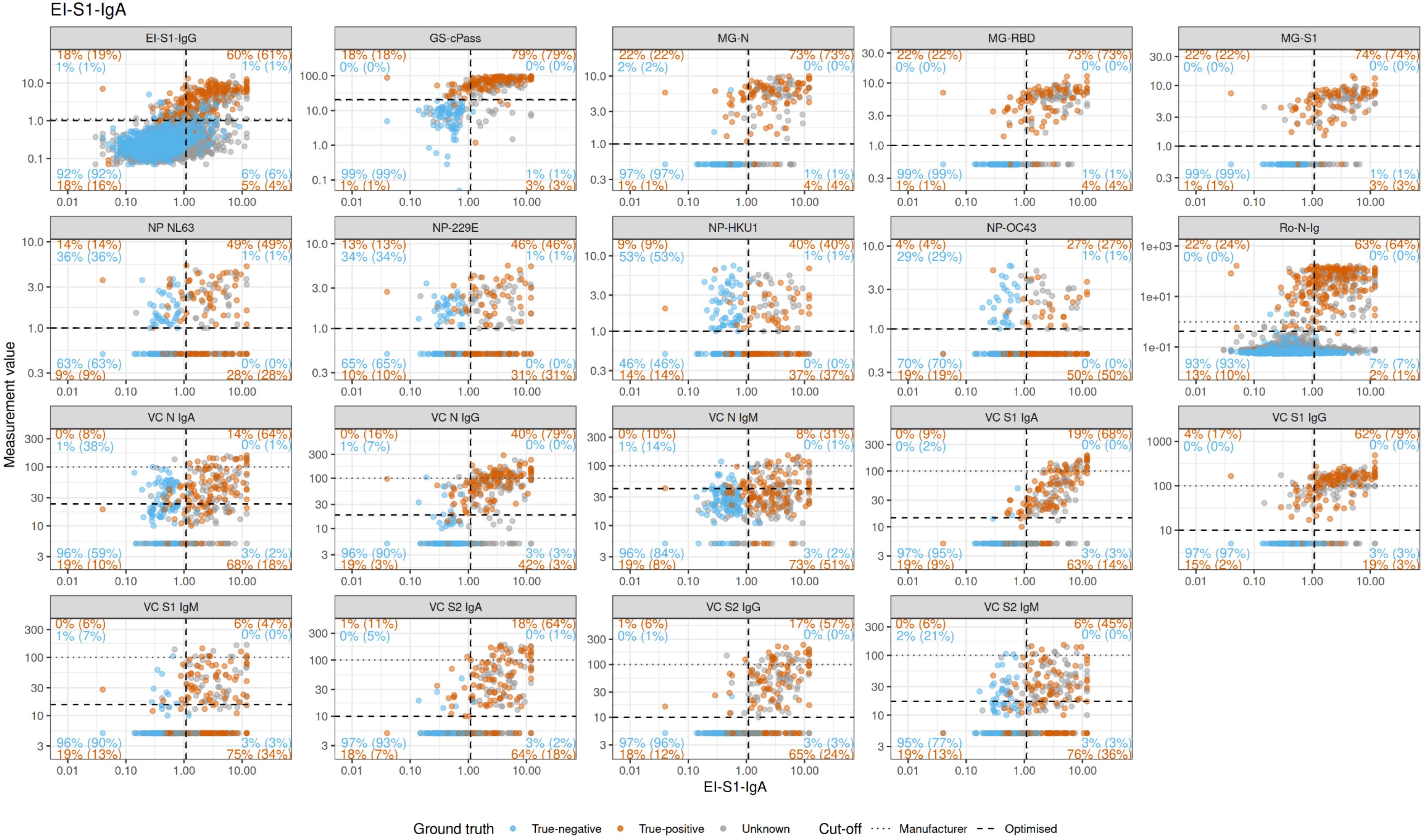
EI-S1-IgA
EI-S1-IgG
GS-cPass
MG-N
MG-RBD
MG-S1
NP NL63
NP-229E
NP-HKU1
NP-OC43
Ro-N-Ig
VC N IgA
VC N IgG
VC N IgM
VC S1 IgA
VC S1 IgG
VC S1 IgM
VC S2 IgA
VC S2 IgG
VC S2 IgM
Measurement value
EI-S1-IgA
Ground truth
True-negative
True-positive
Unknown
Cut-off
Manufacturer
Optimised

Suppl.Fig.7B

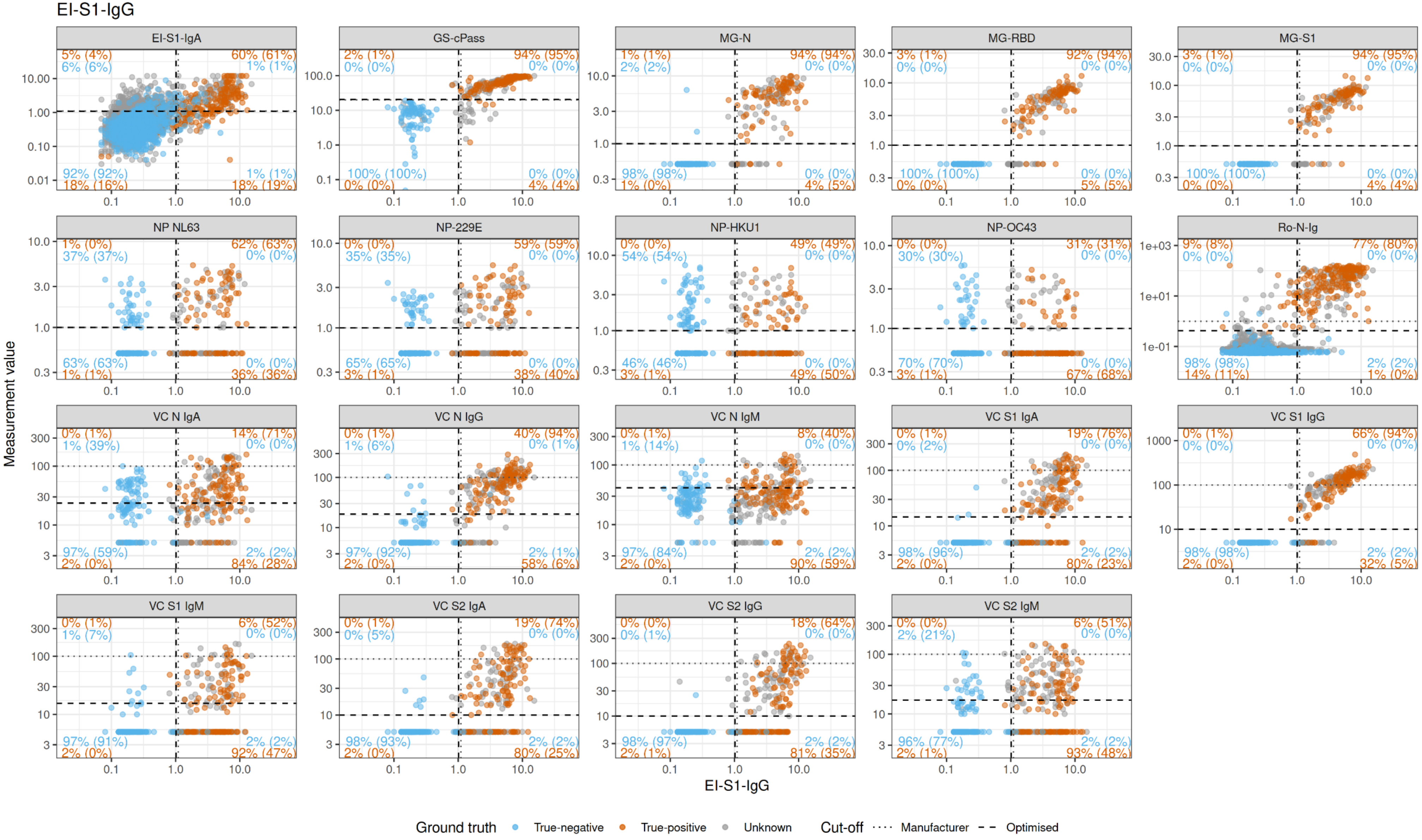

EI-S1-IgG
EI-S1-IgA
GS-cPass
MG-N
MG-RBD
MG-S1
NP NL63
NP-229E
NP-HKU1
NP-OC43
Ro-N-Ig
VC N IgA
VC N IgG
VC N IgM
VC S1 IgA
VC S1 IgG
VC S1 IgM
VC S2 IgA
VC S2 IgG
VC S2 IgM
Measurement value
EI-S1-IgG
Ground truth
True-negative
True-positive
Unknown
Cut-off
Manufacturer
Optimised

Suppl.Fig.7C

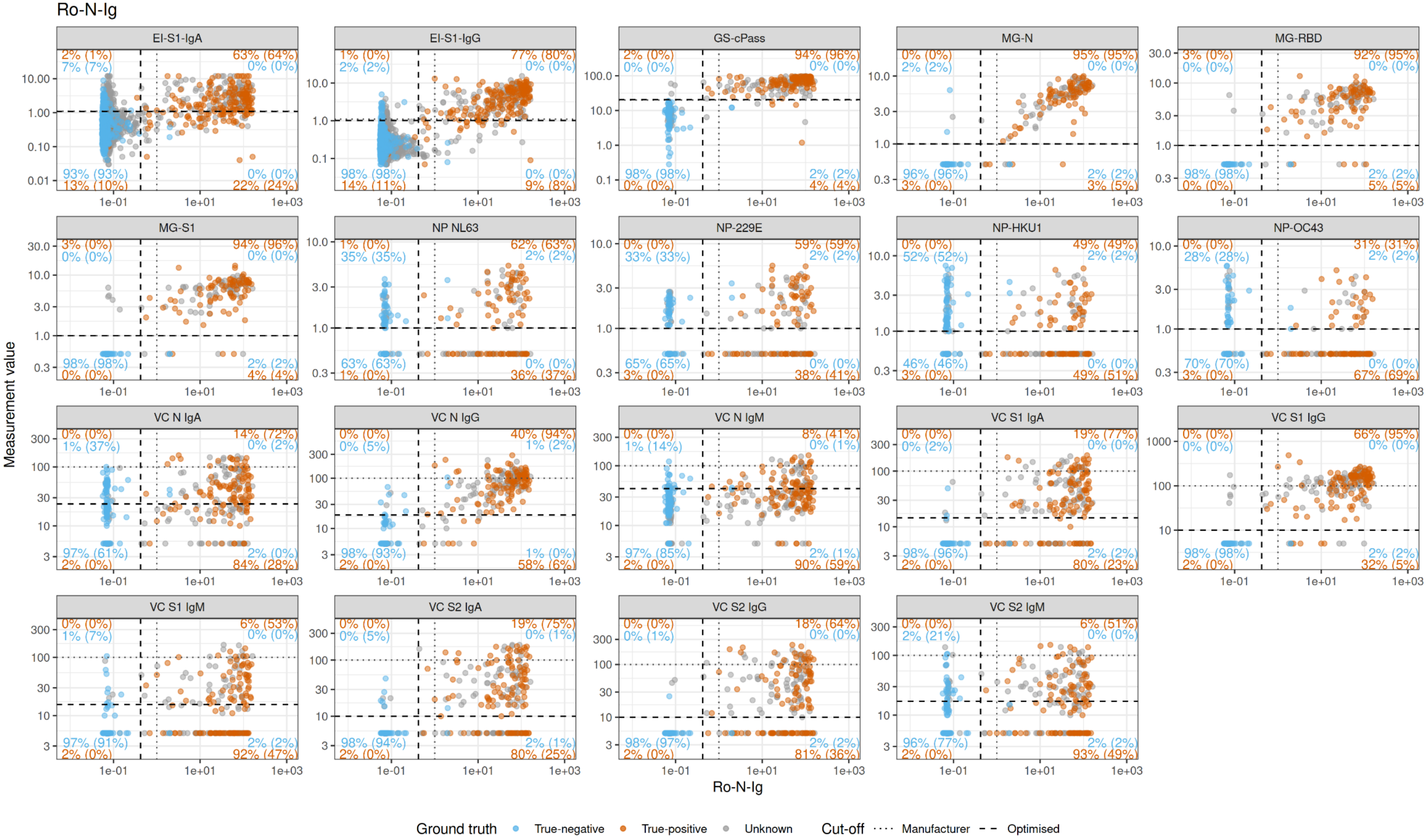
Ro-N-Ig
EI-S1-IgA
2% (1%) 7% (7%) 63% (64%) 0% (0%) 93% (93%) 13% (10%) 0% (0%) 22% (24%)
EI-S1-IgG
1% (0%) 2% (2%) 77% (80%) 0% (0%) 98% (98%) 14% (11%) 0% (0%) 9% (8%)
GS-cPass
2% (0%) 0% (0%) 94% (96%) 0% (0%) 98% (98%) 0% (0%) 2% (2%) 4% (4%)
MG-N
0% (0%) 2% (2%) 95% (95%) 0% (0%) 96% (96%) 3% (0%) 2% (2%) 3% (5%)
MG-RBD
3% (0%) 0% (0%) 92% (95%) 0% (0%) 98% (98%) 0% (0%) 2% (2%) 5% (5%)
MG-S1
3% (0%) 0% (0%) 94% (96%) 0% (0%) 98% (98%) 0% (0%) 2% (2%) 4% (4%)
NP NL63
1% (0%) 35% (35%) 62% (63%) 2% (2%) 63% (63%) 1% (0%) 0% (0%) 36% (37%)
NP-229E
0% (0%) 33% (33%) 59% (59%) 2% (2%) 65% (65%) 3% (0%) 0% (0%) 38% (41%)
NP-HKU1
0% (0%) 52% (52%) 49% (49%) 2% (2%) 46% (46%) 3% (0%) 0% (0%) 49% (51%)
NP-OC43
0% (0%) 28% (28%) 31% (31%) 2% (2%) 70% (70%) 3% (0%) 0% (0%) 67% (69%)
VC N IgA
0% (0%) 1% (37%) 14% (72%) 0% (2%) 97% (61%) 2% (0%) 2% (0%) 84% (28%)
VC N IgG
0% (0%) 0% (5%) 40% (94%) 1% (2%) 98% (93%) 2% (0%) 1% (0%) 58% (6%)
VC N IgM
0% (0%) 1% (14%) 8% (41%) 0% (1%) 97% (85%) 2% (0%) 2% (1%) 90% (59%)
VC S1 IgA
0% (0%) 0% (2%) 19% (77%) 0% (0%) 98% (96%) 2% (0%) 2% (2%) 80% (23%)
VC S1 IgG
0% (0%) 0% (0%) 66% (95%) 0% (0%) 98% (98%) 2% (0%) 2% (2%) 32% (5%)
VC S1 IgM
0% (0%) 1% (7%) 6% (53%) 0% (0%) 97% (91%) 2% (0%) 2% (2%) 92% (47%)
VC S2 IgA
0% (0%) 0% (5%) 19% (75%) 0% (1%) 98% (94%) 2% (0%) 2% (1%) 80% (25%)
VC S2 IgG
0% (0%) 0% (1%) 18% (64%) 0% (0%) 98% (97%) 2% (0%) 2% (2%) 81% (36%)
VC S2 IgM
0% (0%) 2% (21%) 6% (51%) 0% (0%) 96% (77%) 2% (0%) 2% (2%) 93% (49%)
Measurement value
Ro-N-Ig
Ground truth
True-negative
True-positive
Unknown
Cut-off
Manufacturer
Optimised

Supplemental Figure 7: Scatterplots of confirmatory tests vs primary (using optimised and manufacturer's cut-off).

True-negatives in blue, true-positives in orange, unknown SARS-CoV-2 status in grey. Black dotted and dashed lines represent the manufacturer's and the optimised positivity cut-offs. Orange/blue numbers indicate the percentages of true-positives/negatives correctly detected using optimised cut-offs. The orange value above the dotted line represents the percentage of positive test results for the true-positive cohort, the blue number below is the percentage of negatives in the true-negative cohort.

(A) Values obtained with EI-S1-IgA
(B) Values obtained with EI-S1-IgG
(C) Values obtained with Ro-N-Ig

Suppl.Fig.8A

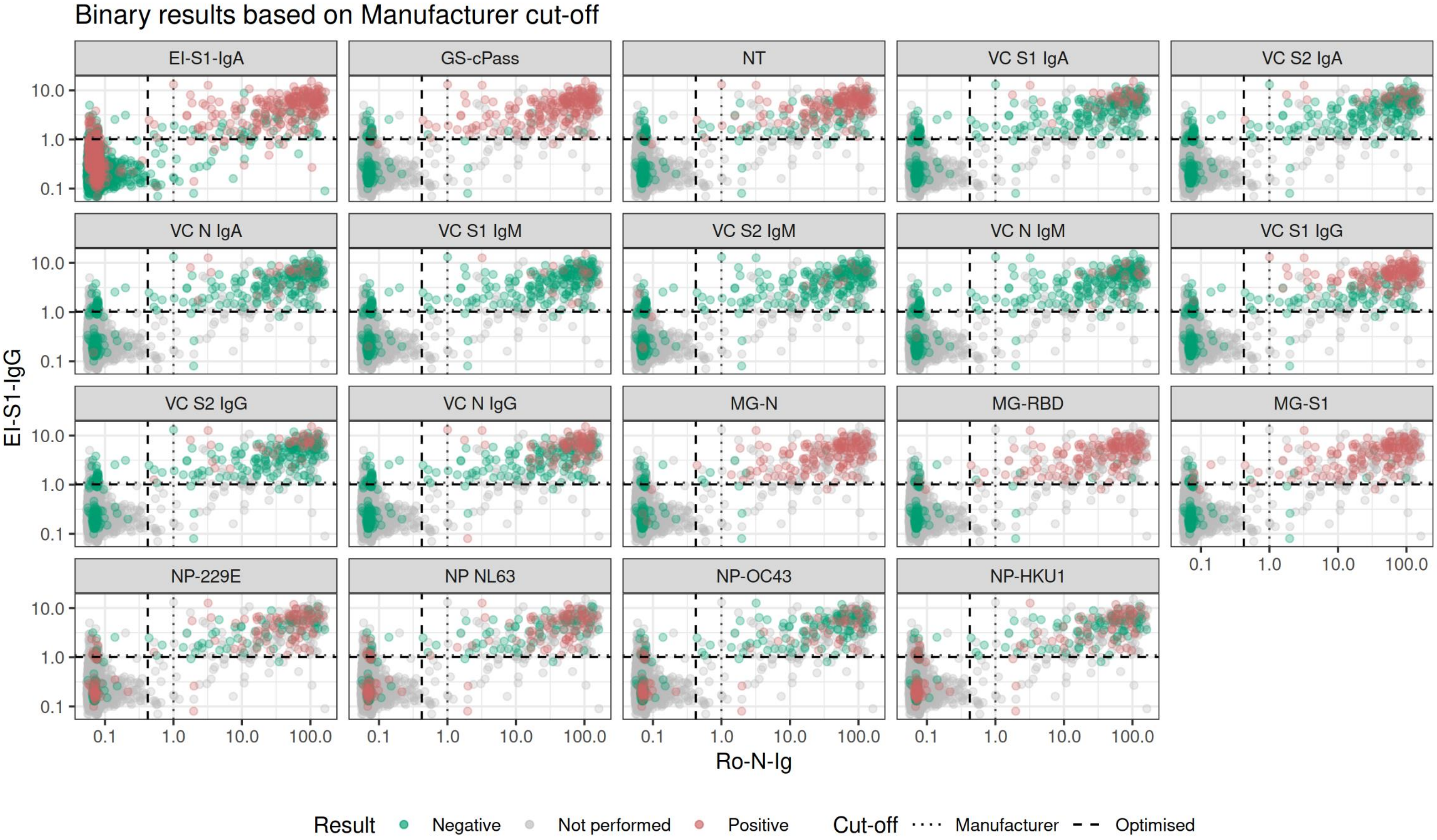
Binary results based on Manufacturer cut-off
EI-S1-IgA
GS-cPass
NT
VC S1 IgA
VC S2 IgA
VC N IgA
VC S1 IgM
VC S2 IgM
VC N IgM
VC S1 IgG
VC S2 IgG
VC N IgG
MG-N
MG-RBD
MG-S1
NP-229E
NP NL63
NP-OC43
NP-HKU1
EI-S1-IgG
10.0
1.0
0.1
0.1
1.0
10.0
100.0
Ro-N-Ig
Result
Negative
Not performed
Positive
Cut-off
Manufacturer
Optimised

Suppl.Fig.8B

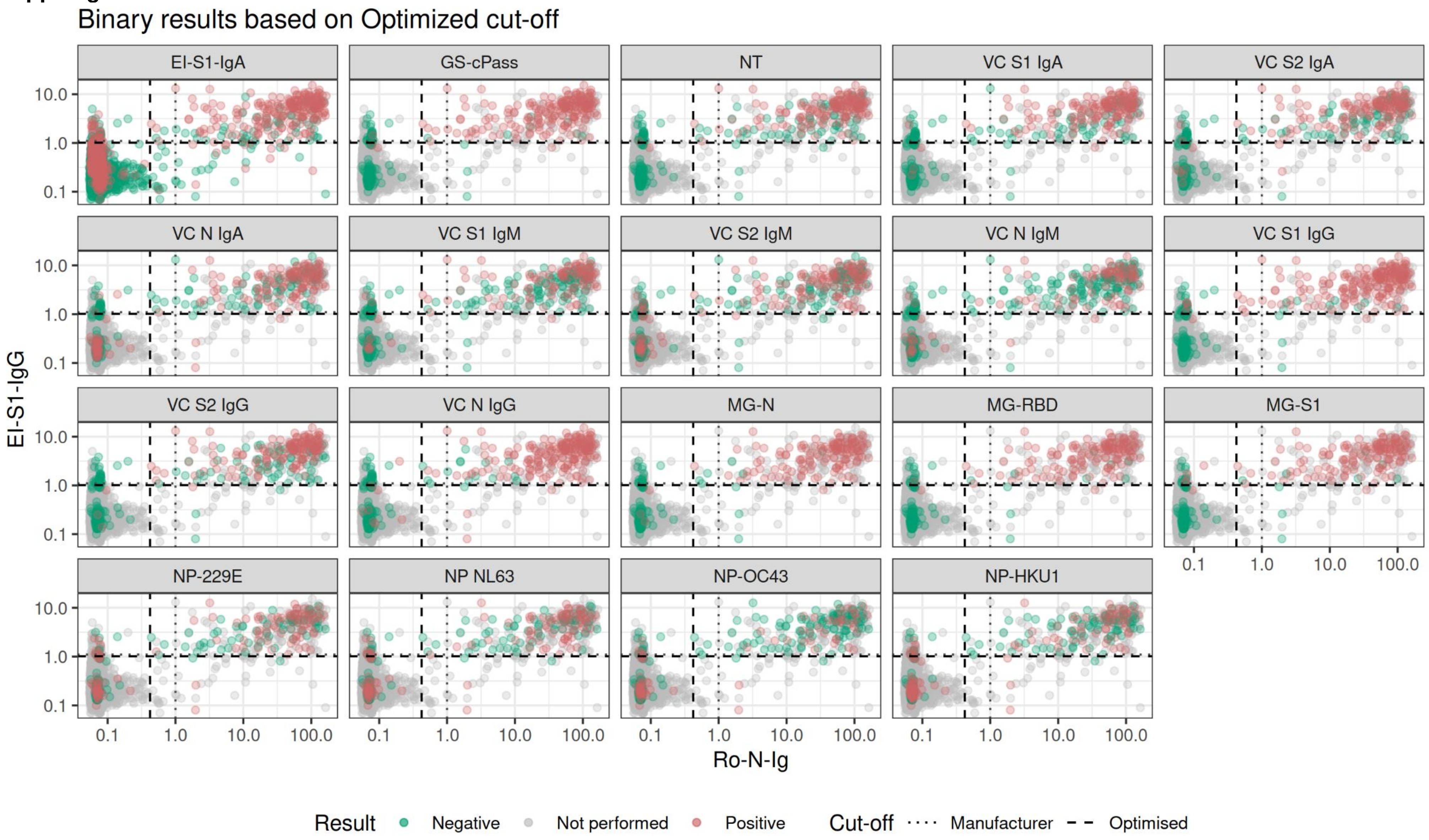

Binary results based on Optimized cut-off
EI-S1-IgA
GS-cPass
NT
VC S1 IgA
VC S2 IgA
VC N IgA
VC S1 IgM
VC S2 IgM
VC N IgM
VC S1 IgG
VC S2 IgG
VC N IgG
MG-N
MG-RBD
MG-S1
NP-229E
NP NL63
NP-OC43
NP-HKU1
EI-S1-IgG
Ro-N-Ig
10.0
1.0
0.1
100.0
Result
Negative
Not performed
Positive
Cut-off
Manufacturer
Optimised

Supplemental Figure 8: Ro-N-Ig vs. EI-S1-IgG showing binary results for the tests
(A) Manufacturer’s cut-off
(B) Optimised cut-off

Suppl.Fig.9A

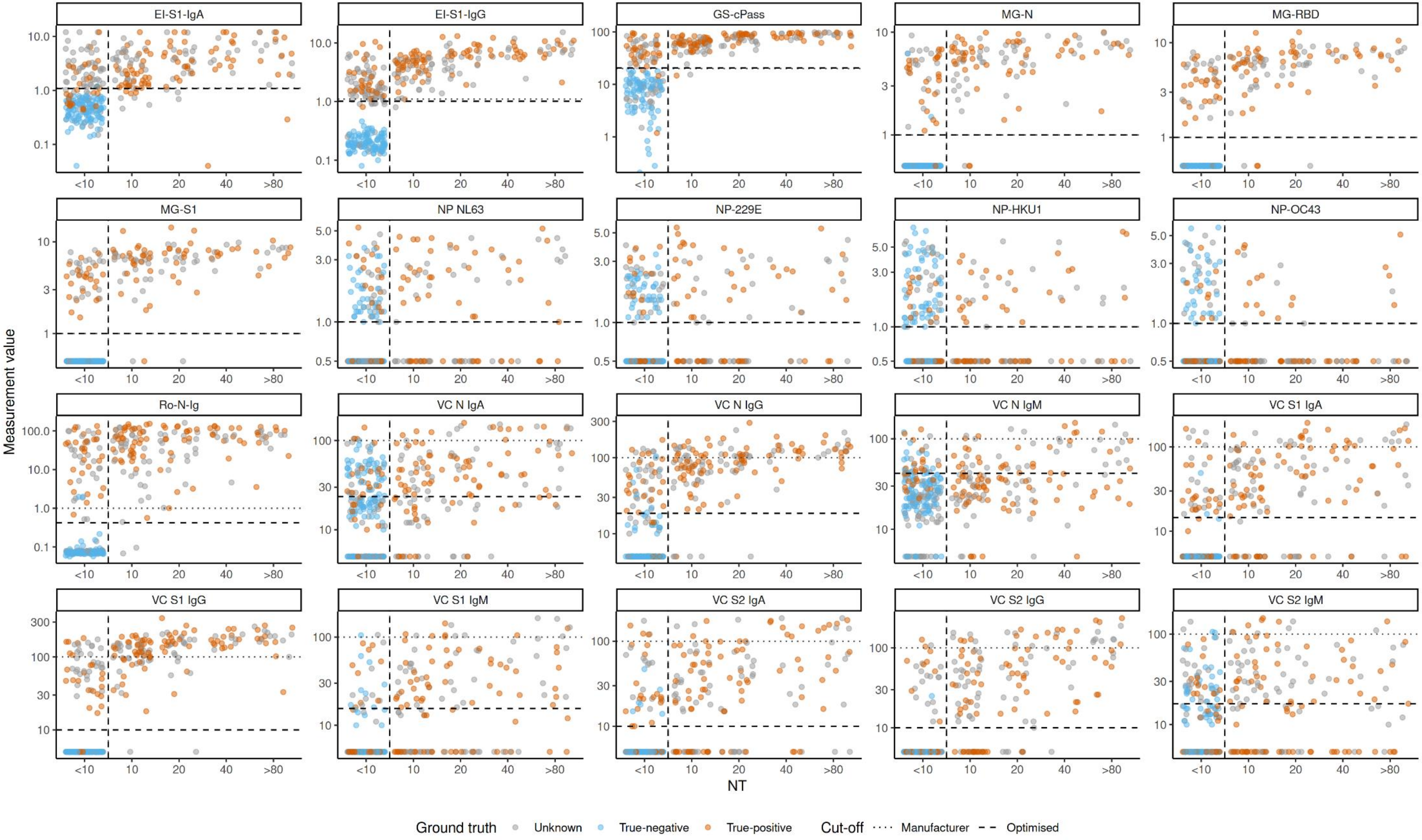
EI-S1-IgA
EI-S1-IgG
GS-cPass
MG-N
MG-RBD
MG-S1
NP NL63
NP-229E
NP-HKU1
NP-OC43
Ro-N-Ig
VC N IgA
VC N IgG
VC N IgM
VC S1 IgA
VC S1 IgG
VC S1 IgM
VC S2 IgA
VC S2 IgG
VC S2 IgM
Measurement value
NT
<10
10
20
40
>80
Ground truth
Unknown
True-negative
True-positive
Cut-off
Manufacturer
Optimised

Suppl.Fig.9B

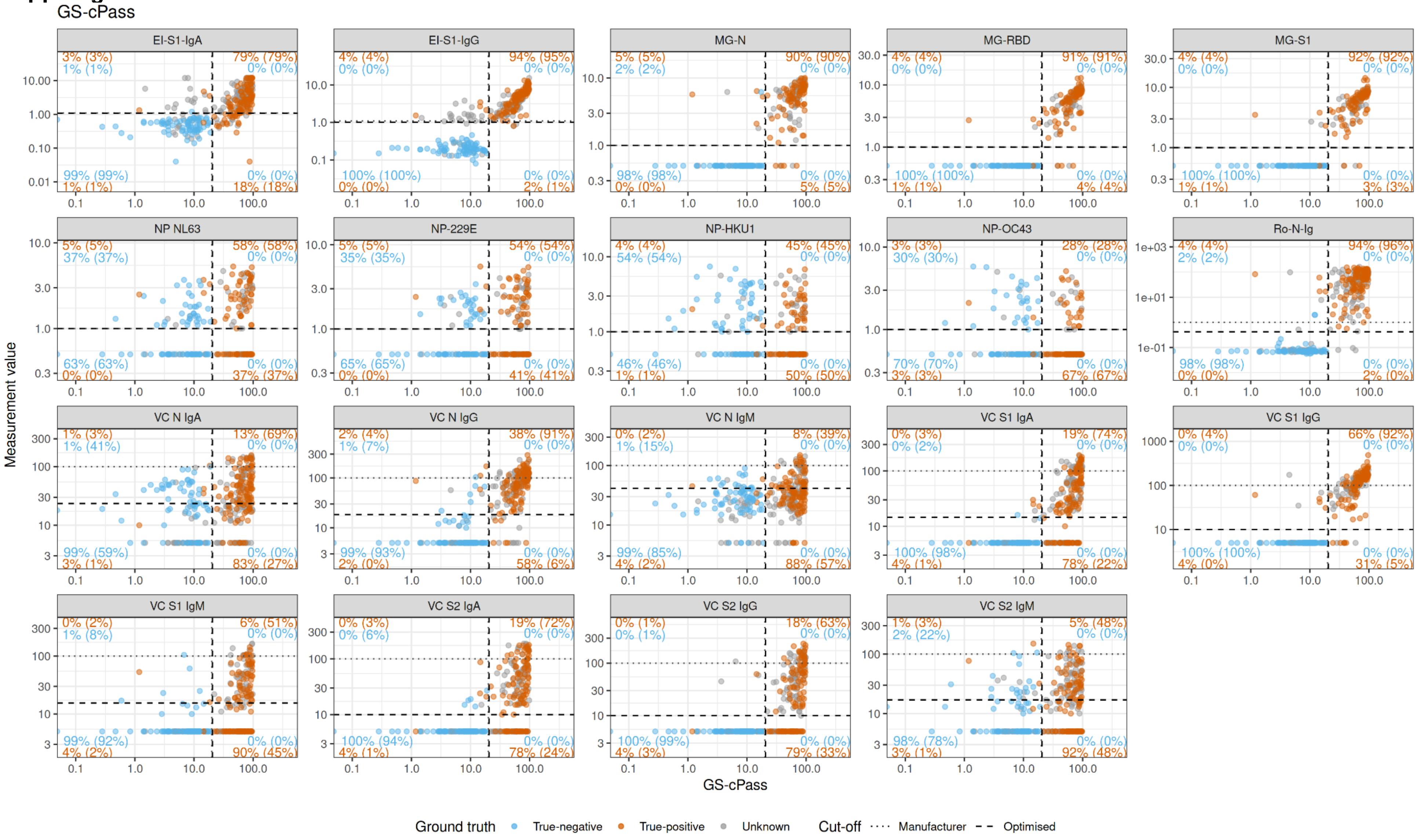
GS-cPass
EI-S1-IgA
EI-S1-IgG
MG-N
MG-RBD
MG-S1
NP NL63
NP-229E
NP-HKU1
NP-OC43
Ro-N-Ig
VC N IgA
VC N IgG
VC N IgM
VC S1 IgA
VC S1 IgG
VC S1 IgM
VC S2 IgA
VC S2 IgG
VC S2 IgM
Measurement value
GS-cPass
Ground truth
True-negative
True-positive
Unknown
Cut-off
Manufacturer
Optimised

Suppl.Fig.9C

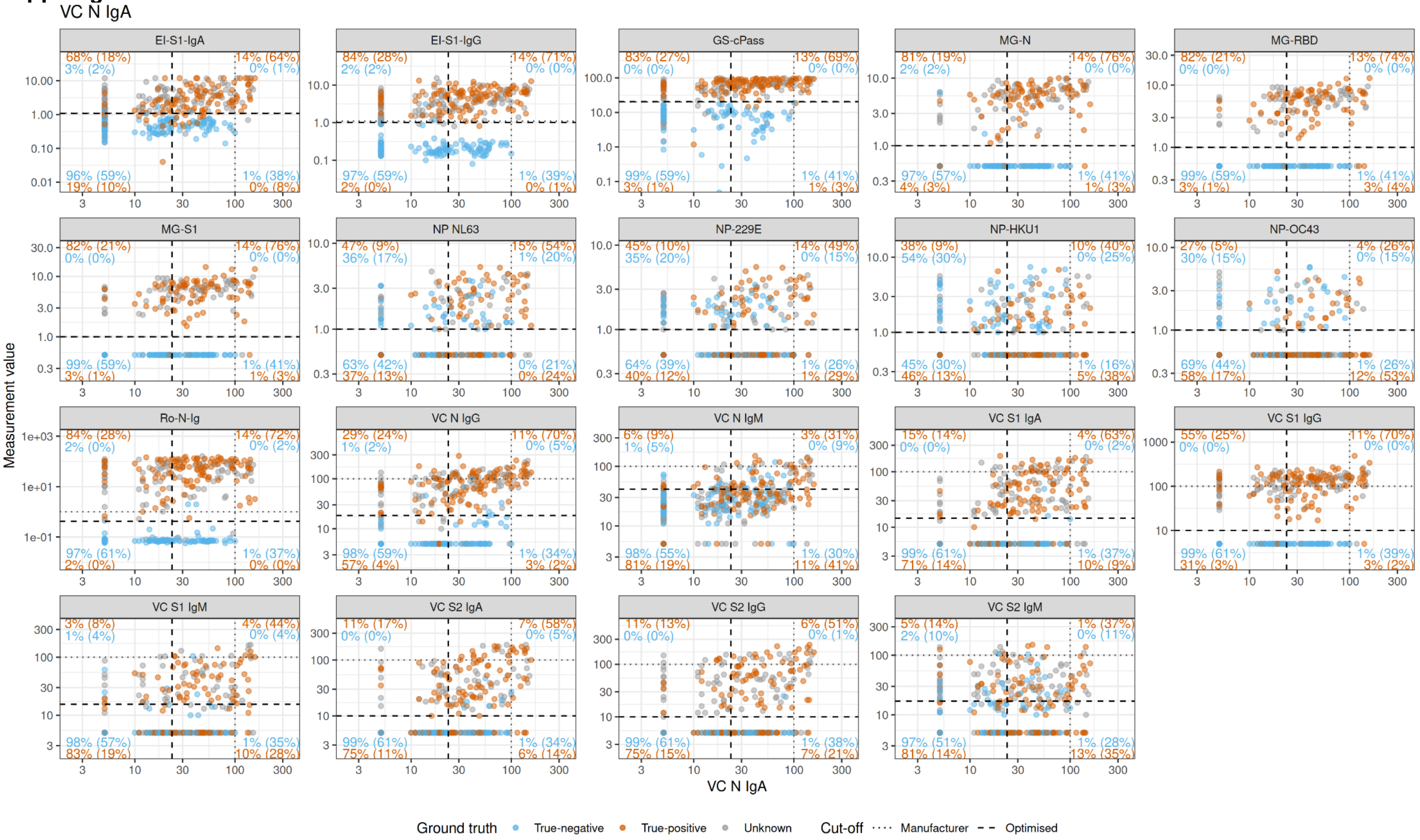
VC N IgA
EI-S1-IgA
EI-S1-IgG
GS-cPass
MG-N
MG-RBD
MG-S1
NP NL63
NP-229E
NP-HKU1
NP-OC43
Ro-N-Ig
VC N IgG
VC N IgM
VC S1 IgA
VC S1 IgG
VC S1 IgM
VC S2 IgA
VC S2 IgG
VC S2 IgM
Measurement value
VC N IgA
Ground truth
True-negative
True-positive
Unknown
Cut-off
Manufacturer
Optimised

Suppl.Fig.9D

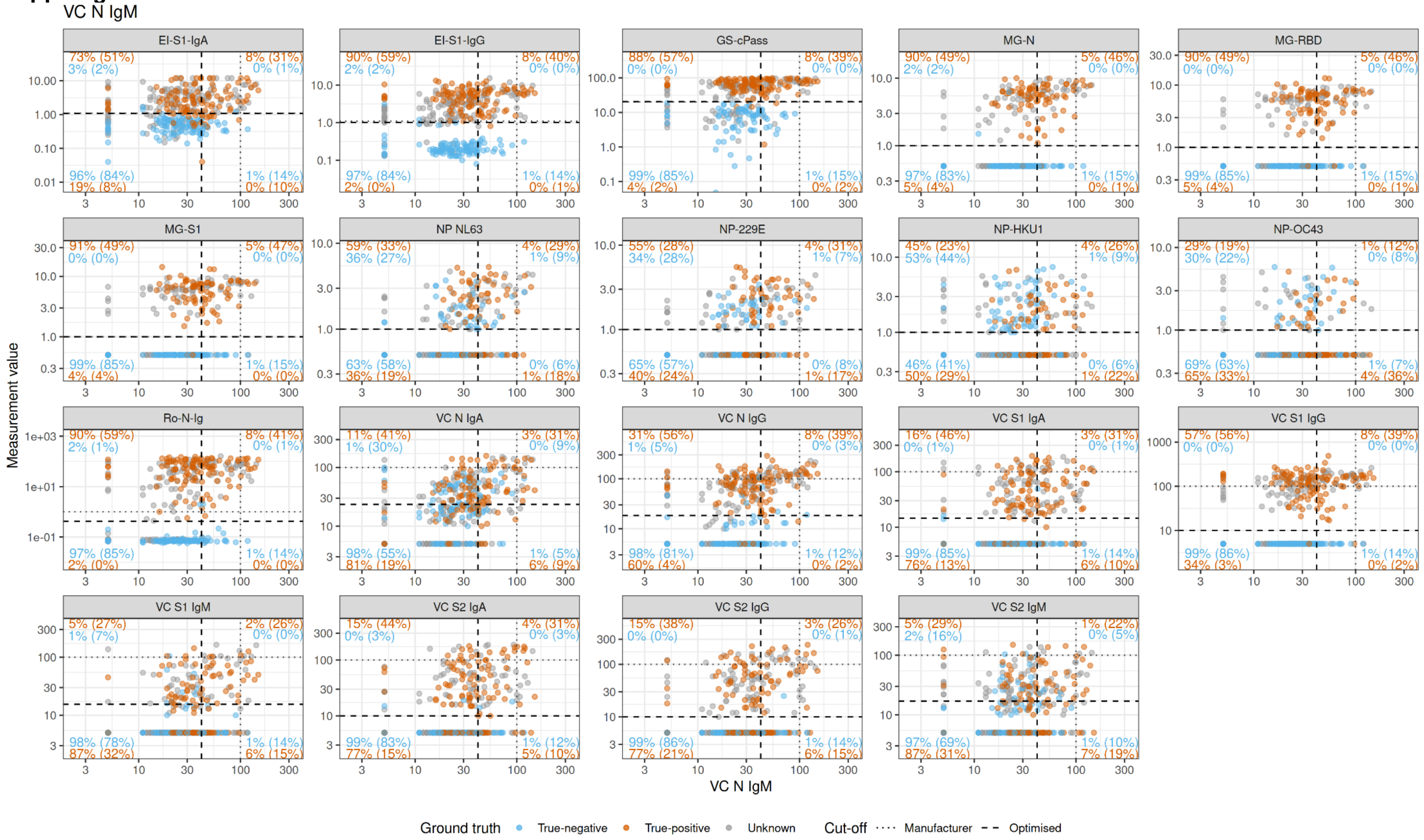
VC N IgM
EI-S1-IgA
EI-S1-IgG
GS-cPass
MG-N
MG-RBD
MG-S1
NP NL63
NP-229E
NP-HKU1
NP-OC43
Ro-N-Ig
VC N IgA
VC N IgG
VC S1 IgA
VC S1 IgG
VC S1 IgM
VC S2 IgA
VC S2 IgG
VC S2 IgM
Measurement value
VC N IgM
Ground truth
True-negative
True-positive
Unknown
Cut-off
Manufacturer
Optimised

Suppl.Fig.9E

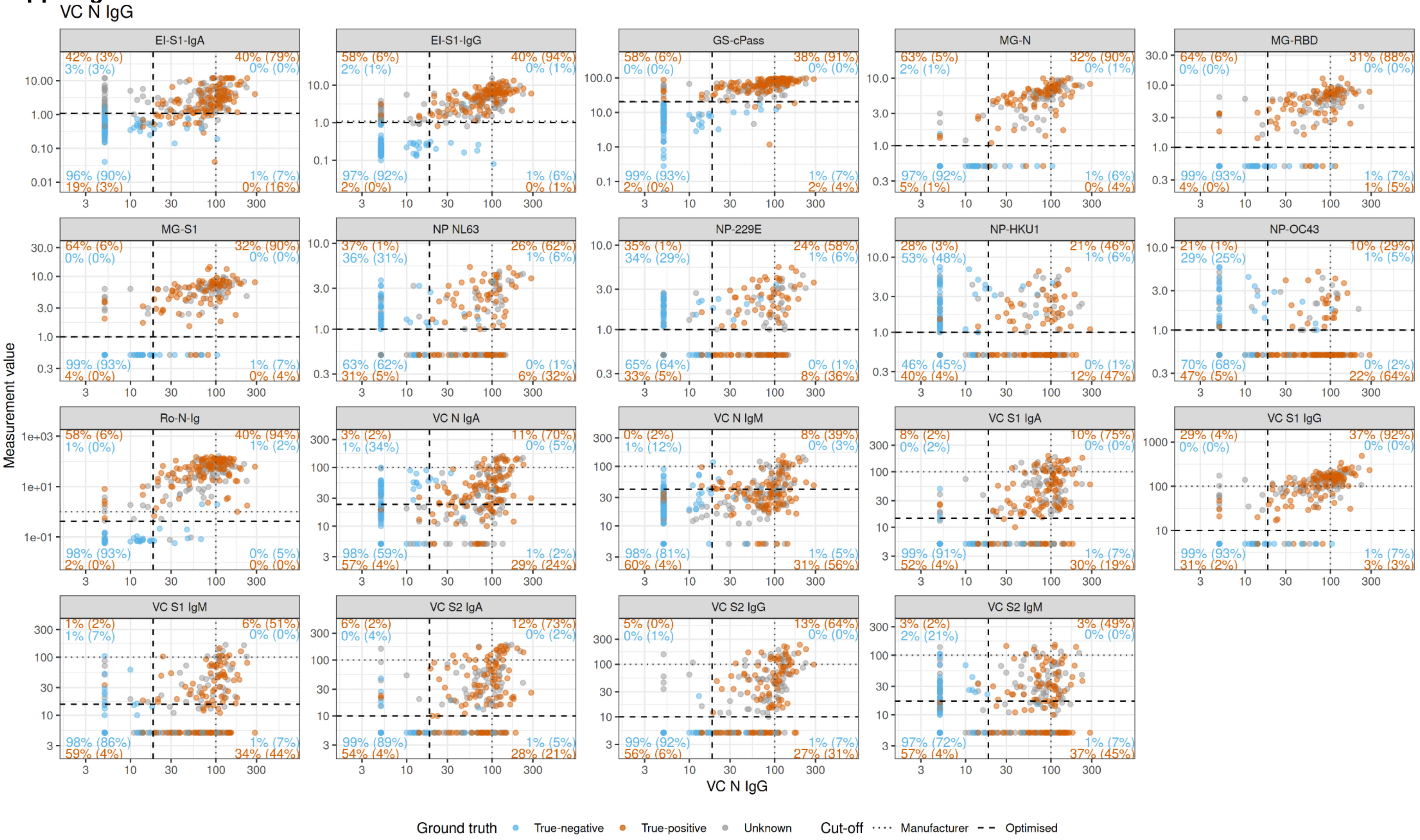
VC N IgG
EI-S1-IgA
EI-S1-IgG
GS-cPass
MG-N
MG-RBD
MG-S1
NP NL63
NP-229E
NP-HKU1
NP-OC43
Ro-N-Ig
VC N IgA
VC N IgM
VC S1 IgA
VC S1 IgG
VC S1 IgM
VC S2 IgA
VC S2 IgG
VC S2 IgM
Measurement value
VC N IgG
Ground truth
True-negative
True-positive
Unknown
Cut-off
Manufacturer
Optimised

Suppl.Fig.9F

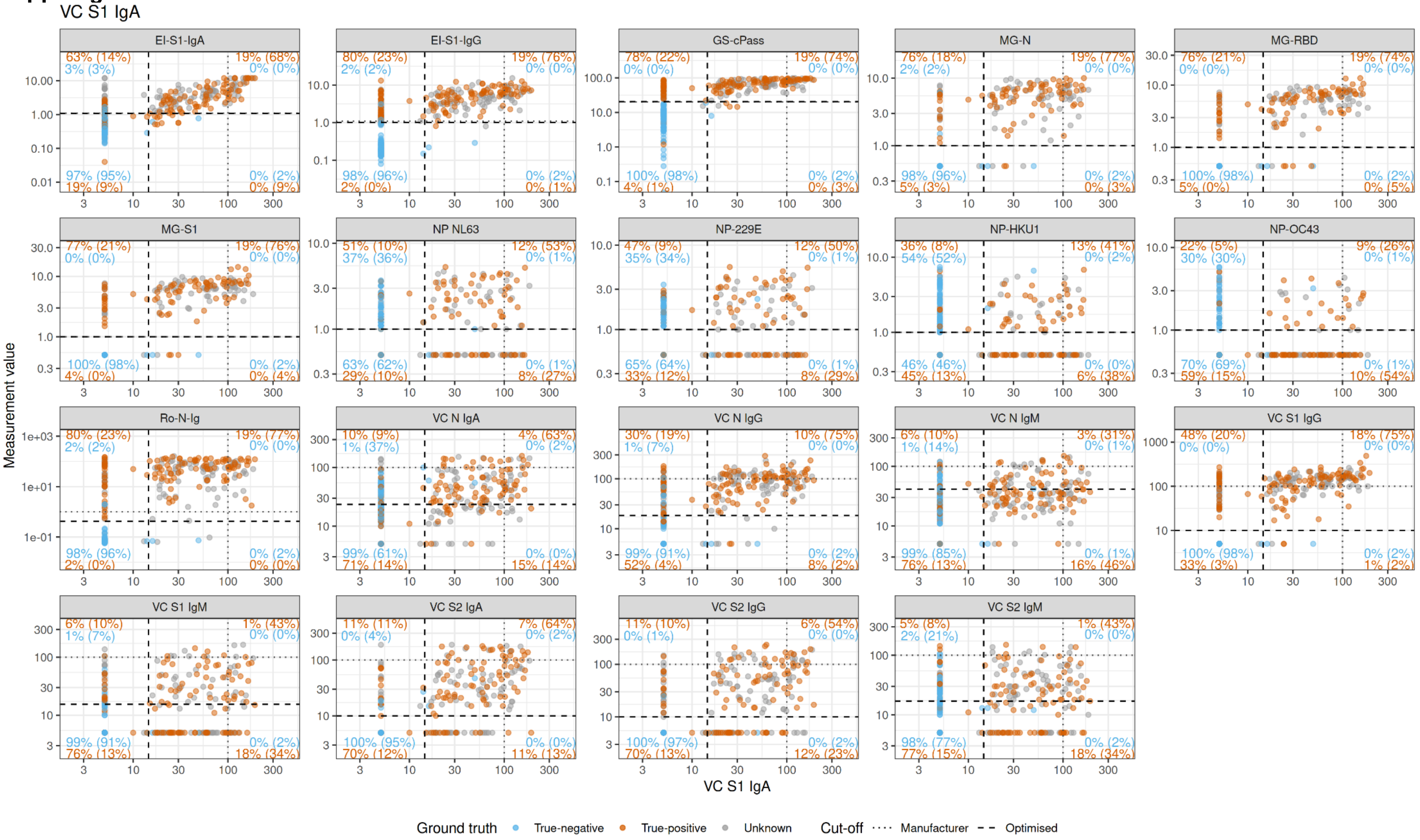
VC S1 IgA
EI-S1-IgA
EI-S1-IgG
GS-cPass
MG-N
MG-RBD
MG-S1
NP NL63
NP-229E
NP-HKU1
NP-OC43
Ro-N-Ig
VC N IgA
VC N IgG
VC N IgM
VC S1 IgG
VC S1 IgM
VC S2 IgA
VC S2 IgG
VC S2 IgM
Measurement value
VC S1 IgA
Ground truth
True-negative
True-positive
Unknown
Cut-off
Manufacturer
Optimised

Suppl.Fig.9G

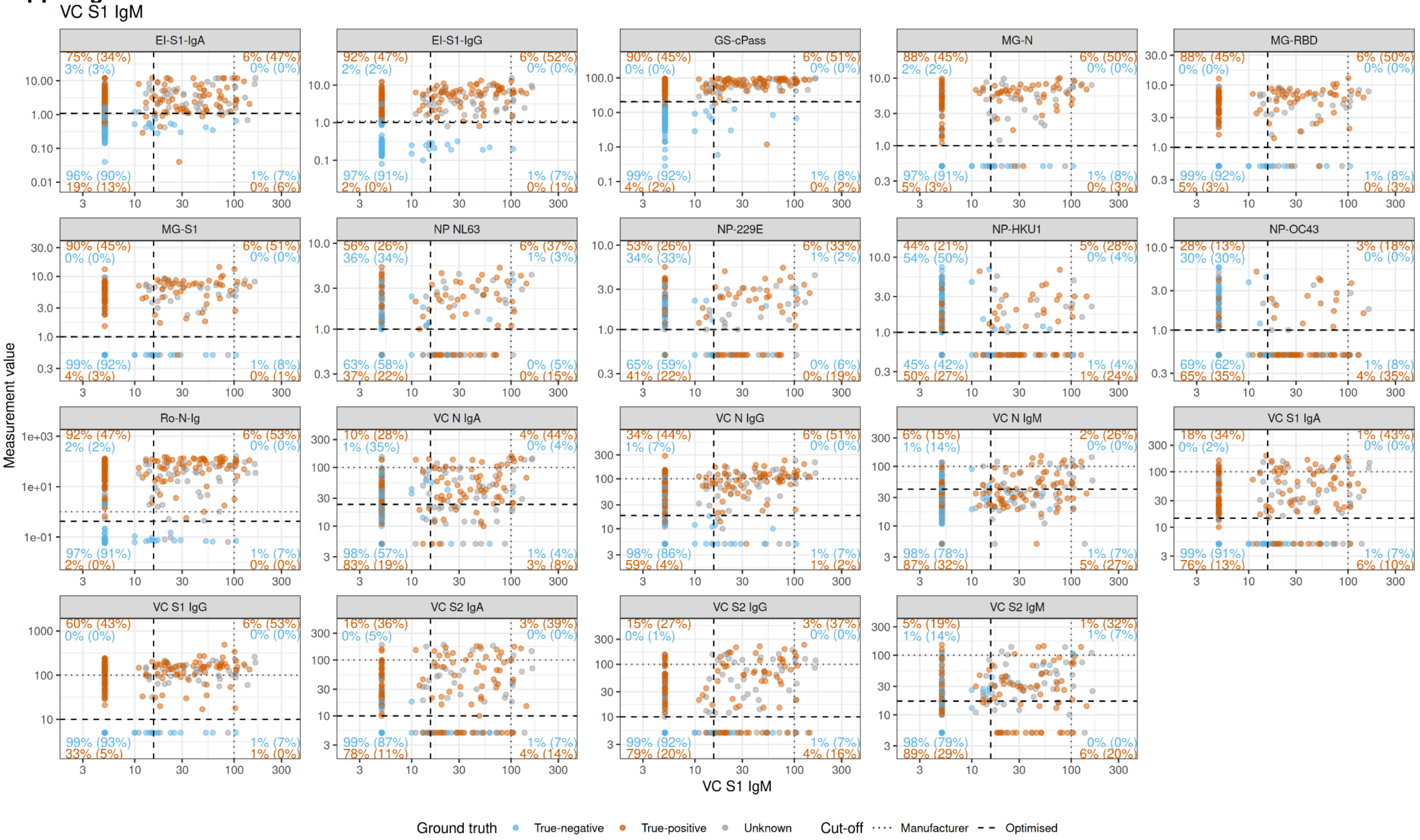
VC S1 IgM
EI-S1-IgA
EI-S1-IgG
GS-cPass
MG-N
MG-RBD
MG-S1
NP NL63
NP-229E
NP-HKU1
NP-OC43
Ro-N-Ig
VC N IgA
VC N IgG
VC N IgM
VC S1 IgA
VC S1 IgG
VC S2 IgA
VC S2 IgG
VC S2 IgM
Measurement value
VC S1 IgM
Ground truth
True-negative
True-positive
Unknown
Cut-off
Manufacturer
Optimised

Suppl.Fig.9H

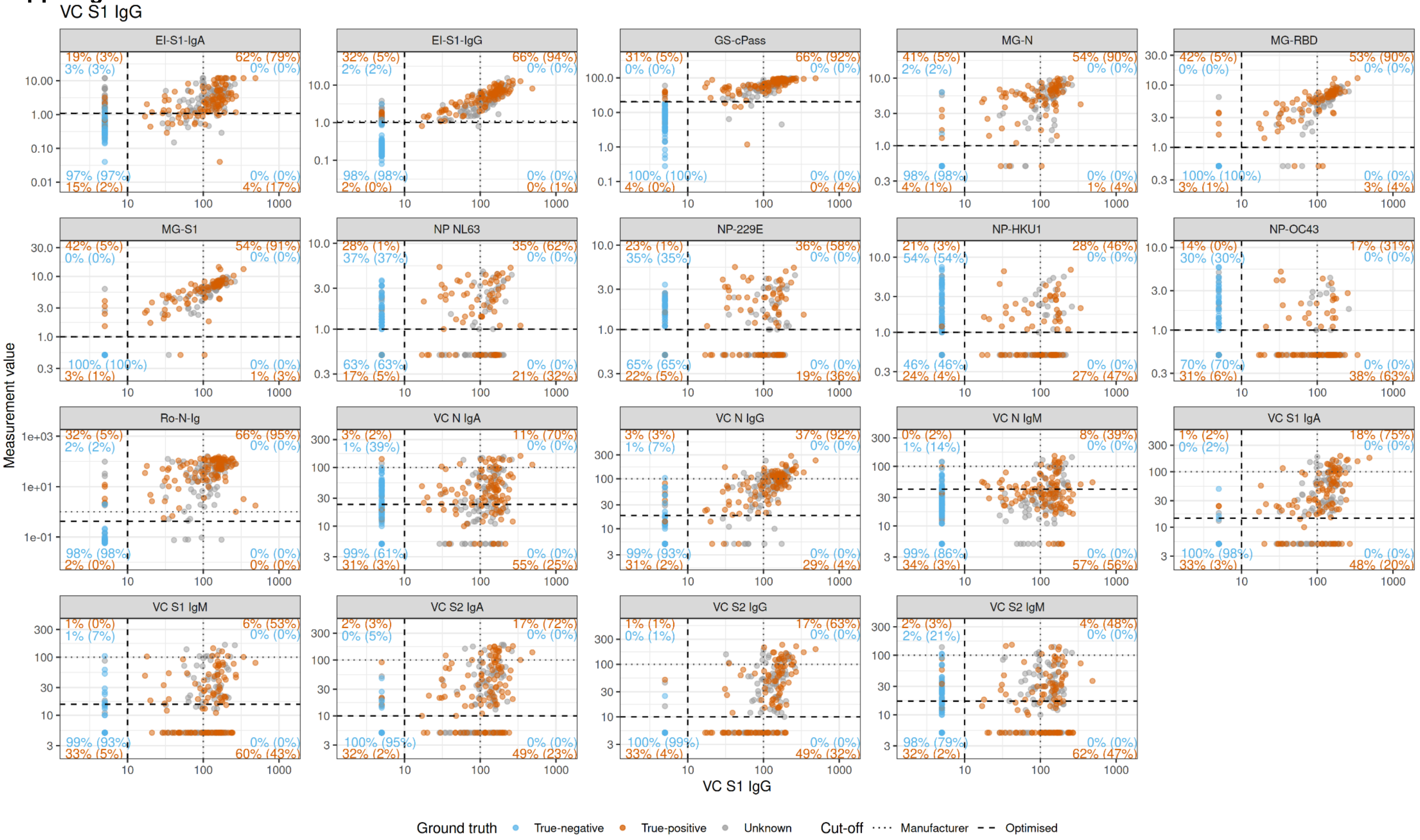
VC S1 IgG
EI-S1-IgA
EI-S1-IgG
GS-cPass
MG-N
MG-RBD
MG-S1
NP NL63
NP-229E
NP-HKU1
NP-OC43
Ro-N-Ig
VC N IgA
VC N IgG
VC N IgM
VC S1 IgA
VC S1 IgM
VC S2 IgA
VC S2 IgG
VC S2 IgM
Measurement value
VC S1 IgG
Ground truth
True-negative
True-positive
Unknown
Cut-off
Manufacturer
Optimised

Suppl.Fig.9I

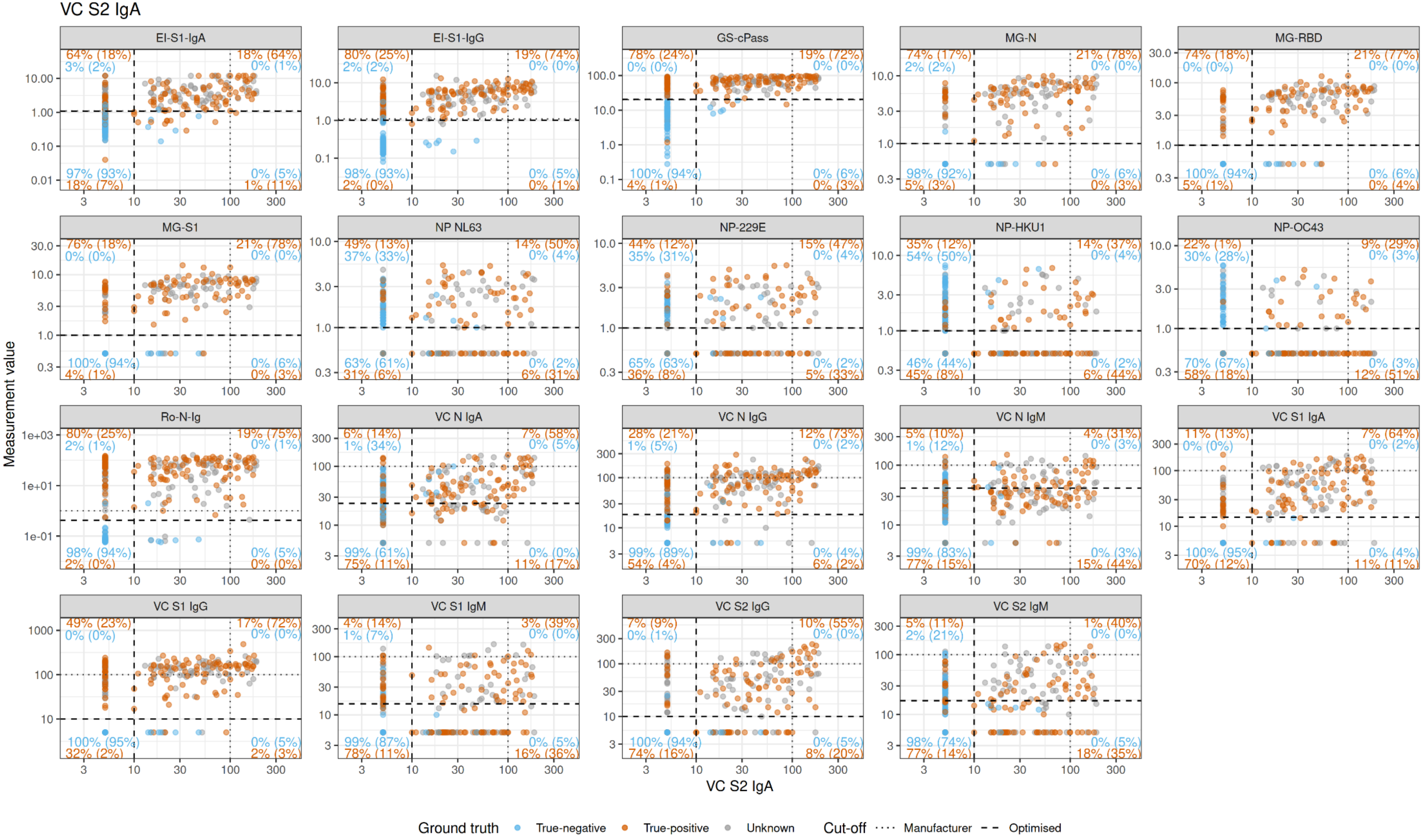
VC S2 IgA
EI-S1-IgA
EI-S1-IgG
GS-cPass
MG-N
MG-RBD
MG-S1
NP NL63
NP-229E
NP-HKU1
NP-OC43
Ro-N-Ig
VC N IgA
VC N IgG
VC N IgM
VC S1 IgA
VC S1 IgG
VC S1 IgM
VC S2 IgG
VC S2 IgM
Measurement value
VC S2 IgA
Ground truth
True-negative
True-positive
Unknown
Cut-off
Manufacturer
Optimised

Suppl.Fig.9J

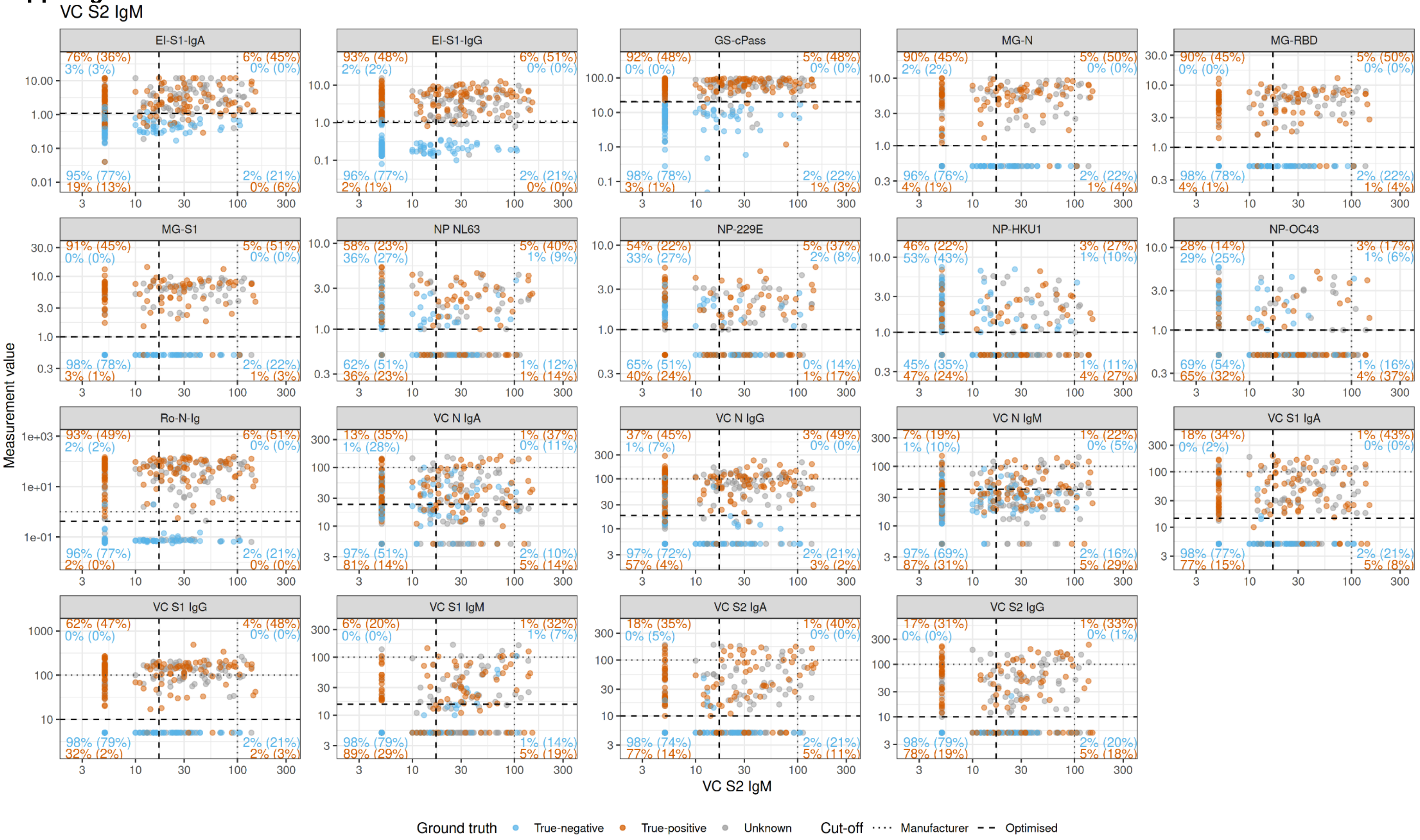

VC S2 IgM
EI-S1-IgA
EI-S1-IgG
GS-cPass
MG-N
MG-RBD
MG-S1
NP NL63
NP-229E
NP-HKU1
NP-OC43
Ro-N-Ig
VC N IgA
VC N IgG
VC N IgM
VC S1 IgA
VC S1 IgG
VC S1 IgM
VC S2 IgA
VC S2 IgG
Measurement value
VC S2 IgM
Ground truth
True-negative
True-positive
Unknown
Cut-off
Manufacturer
Optimised

Suppl.Fig.9K

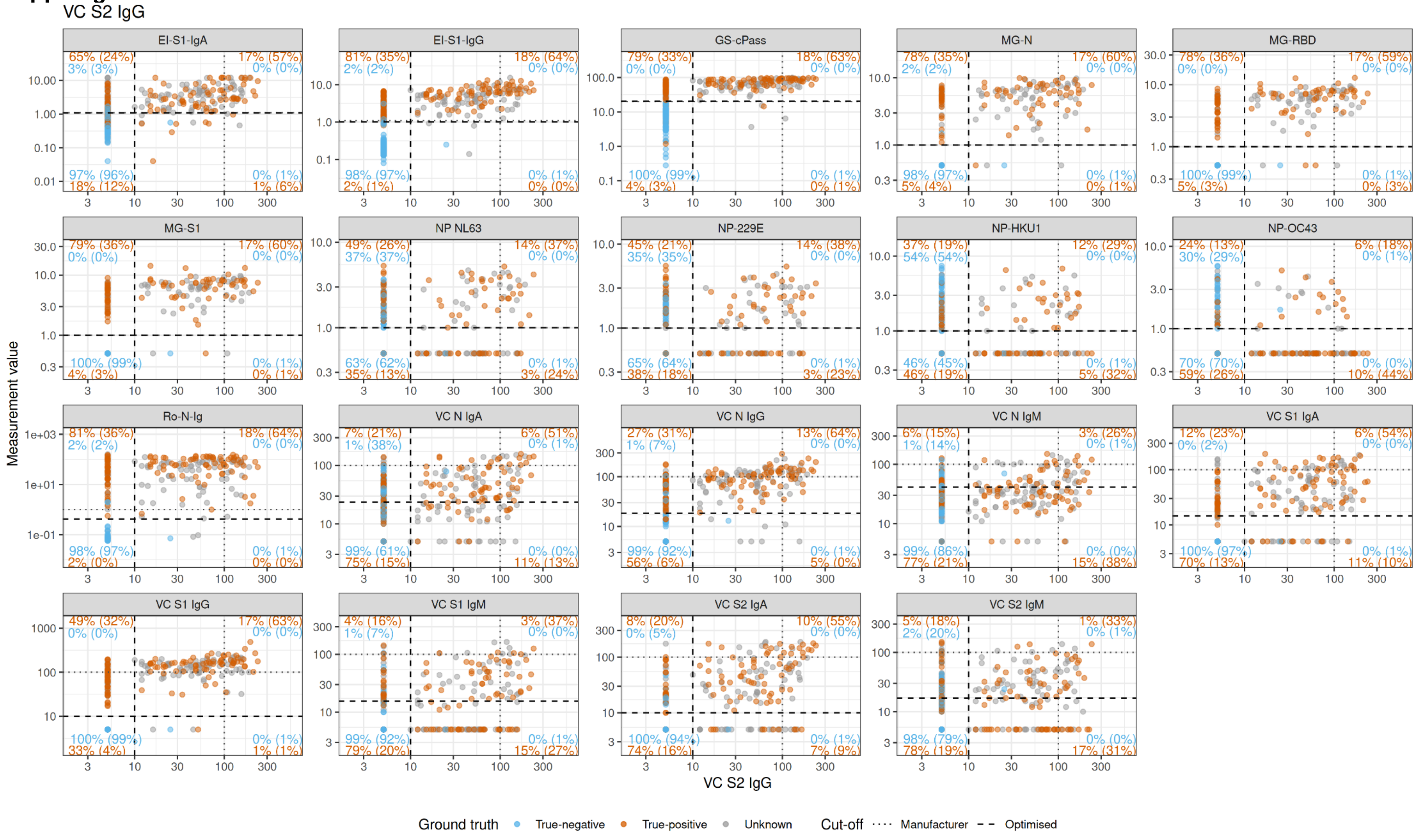
VC S2 IgG
EI-S1-IgA
EI-S1-IgG
GS-cPass
MG-N
MG-RBD
MG-S1
NP NL63
NP-229E
NP-HKU1
NP-OC43
Ro-N-Ig
VC N IgA
VC N IgG
VC N IgM
VC S1 IgA
VC S1 IgG
VC S1 IgM
VC S2 IgA
VC S2 IgM
Measurement value
VC S2 IgG
Ground truth
True-negative
True-positive
Unknown
Cut-off
Manufacturer
Optimised

Suppl.Fig.9L

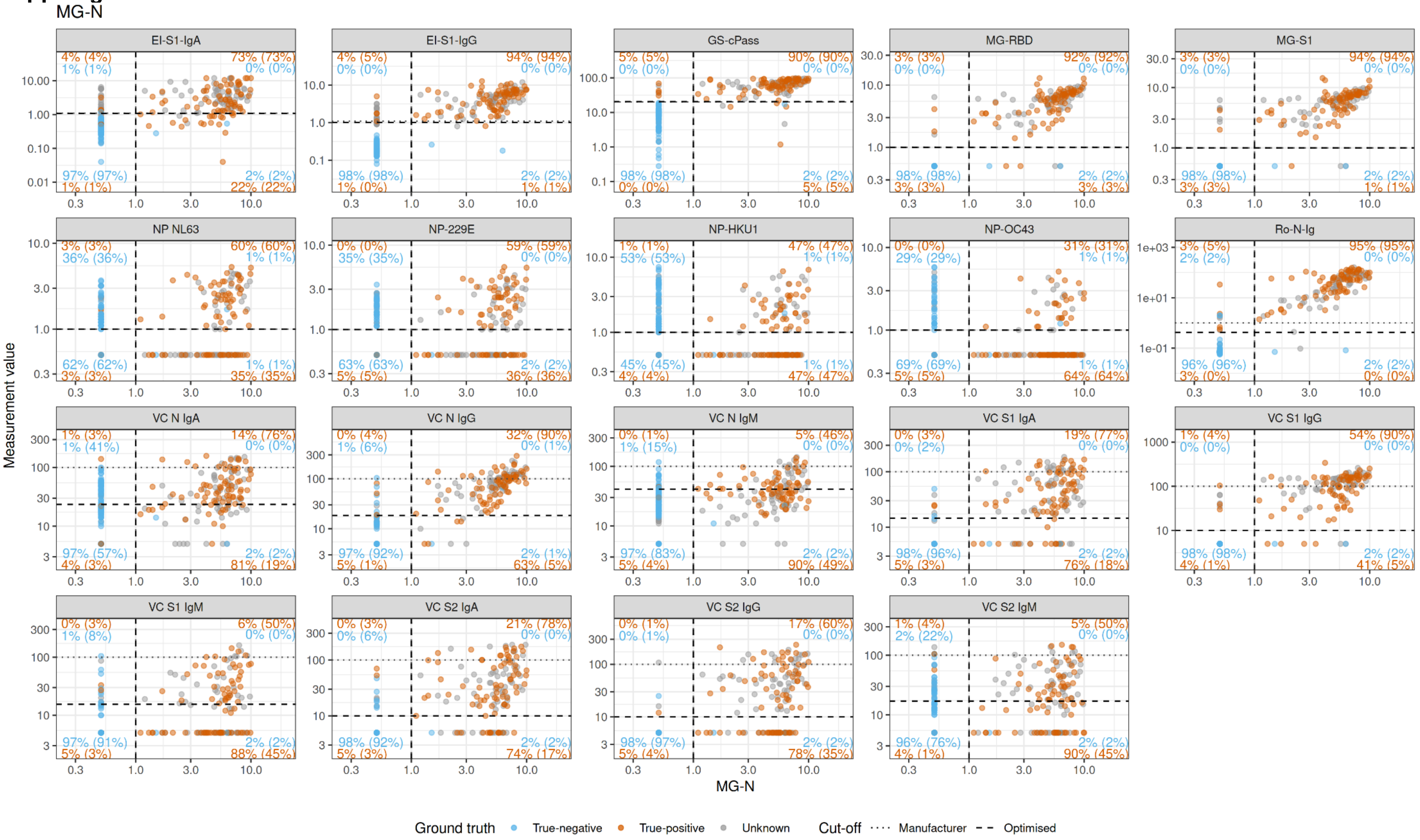
MG-N
EI-S1-IgA
4% (4%)
1% (1%)
73% (73%)
0% (0%)
97% (97%)
1% (1%)
2% (2%)
22% (22%)
EI-S1-IgG
4% (5%)
0% (0%)
94% (94%)
0% (0%)
98% (98%)
1% (0%)
2% (2%)
1% (1%)
GS-cPass
5% (5%)
0% (0%)
90% (90%)
0% (0%)
98% (98%)
0% (0%)
2% (2%)
5% (5%)
MG-RBD
3% (3%)
0% (0%)
92% (92%)
0% (0%)
98% (98%)
3% (3%)
2% (2%)
3% (3%)
MG-S1
3% (3%)
0% (0%)
94% (94%)
0% (0%)
98% (98%)
3% (3%)
2% (2%)
1% (1%)
NP NL63
3% (3%)
36% (36%)
60% (60%)
1% (1%)
62% (62%)
3% (3%)
1% (1%)
35% (35%)
NP-229E
0% (0%)
35% (35%)
59% (59%)
0% (0%)
63% (63%)
5% (5%)
2% (2%)
36% (36%)
NP-HKU1
1% (1%)
53% (53%)
47% (47%)
1% (1%)
45% (45%)
4% (4%)
1% (1%)
47% (47%)
NP-OC43
0% (0%)
29% (29%)
31% (31%)
1% (1%)
69% (69%)
5% (5%)
1% (1%)
64% (64%)
Ro-N-Ig
3% (5%)
2% (2%)
95% (95%)
0% (0%)
96% (96%)
3% (0%)
2% (2%)
0% (0%)
VC N IgA
1% (3%)
1% (41%)
14% (76%)
0% (0%)
97% (57%)
4% (3%)
2% (2%)
81% (19%)
VC N IgG
0% (4%)
1% (6%)
32% (90%)
0% (1%)
97% (92%)
5% (1%)
2% (1%)
63% (5%)
VC N IgM
0% (1%)
1% (15%)
5% (46%)
0% (0%)
97% (83%)
5% (4%)
2% (2%)
90% (49%)
VC S1 IgA
0% (3%)
0% (2%)
19% (77%)
0% (0%)
98% (96%)
5% (3%)
2% (2%)
76% (18%)
VC S1 IgG
1% (4%)
0% (0%)
54% (90%)
0% (0%)
98% (98%)
4% (1%)
2% (2%)
41% (5%)
VC S1 IgM
0% (3%)
1% (8%)
6% (50%)
0% (0%)
97% (91%)
5% (3%)
2% (2%)
88% (45%)
VC S2 IgA
0% (3%)
0% (6%)
21% (78%)
0% (0%)
98% (92%)
5% (3%)
2% (2%)
74% (17%)
VC S2 IgG
0% (1%)
0% (1%)
17% (60%)
0% (0%)
98% (97%)
5% (4%)
2% (2%)
78% (35%)
VC S2 IgM
1% (4%)
2% (22%)
5% (50%)
0% (0%)
96% (76%)
4% (1%)
2% (2%)
90% (45%)
Measurement value
MG-N
Ground truth
True-negative
True-positive
Unknown
Cut-off
Manufacturer
Optimised

Suppl.Fig.9M

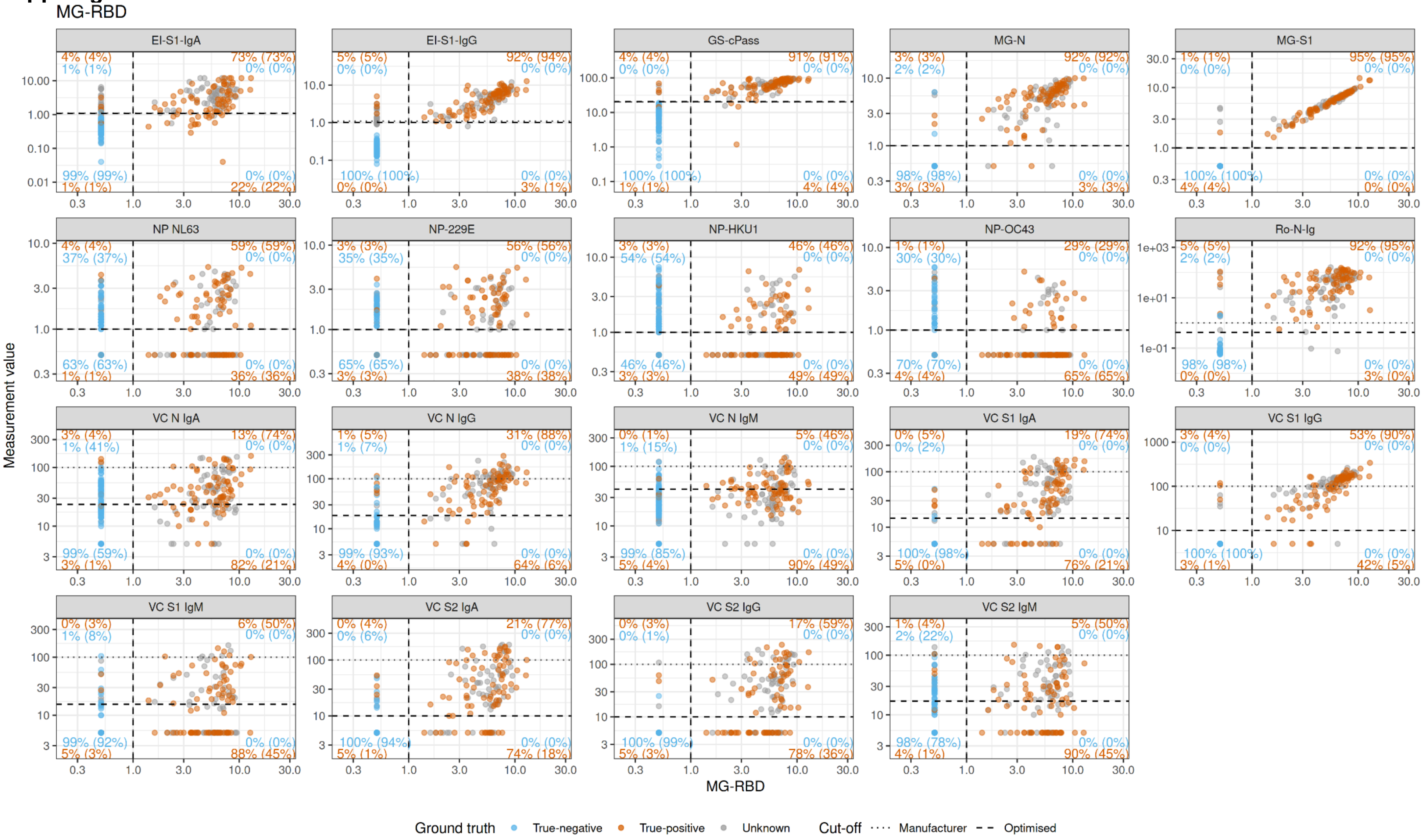
MG-RBD
EI-S1-IgA
4% (4%)
1% (1%)
73% (73%)
0% (0%)
99% (99%)
1% (1%)
0% (0%)
22% (22%)
EI-S1-IgG
5% (5%)
0% (0%)
92% (94%)
0% (0%)
100% (100%)
0% (0%)
0% (0%)
3% (1%)
GS-cPass
4% (4%)
0% (0%)
91% (91%)
0% (0%)
100% (100%)
1% (1%)
0% (0%)
4% (4%)
MG-N
3% (3%)
2% (2%)
92% (92%)
0% (0%)
98% (98%)
3% (3%)
0% (0%)
3% (3%)
MG-S1
1% (1%)
0% (0%)
95% (95%)
0% (0%)
100% (100%)
4% (4%)
0% (0%)
0% (0%)
NP NL63
4% (4%)
37% (37%)
59% (59%)
0% (0%)
63% (63%)
1% (1%)
0% (0%)
36% (36%)
NP-229E
3% (3%)
35% (35%)
56% (56%)
0% (0%)
65% (65%)
3% (3%)
0% (0%)
38% (38%)
NP-HKU1
3% (3%)
54% (54%)
46% (46%)
0% (0%)
46% (46%)
3% (3%)
0% (0%)
49% (49%)
NP-OC43
1% (1%)
30% (30%)
29% (29%)
0% (0%)
70% (70%)
4% (4%)
0% (0%)
65% (65%)
Ro-N-Ig
5% (5%)
2% (2%)
92% (95%)
0% (0%)
98% (98%)
0% (0%)
0% (0%)
3% (0%)
VC N IgA
3% (4%)
1% (41%)
13% (74%)
0% (0%)
99% (59%)
3% (1%)
0% (0%)
82% (21%)
VC N IgG
1% (5%)
1% (7%)
31% (88%)
0% (0%)
99% (93%)
4% (0%)
0% (0%)
64% (6%)
VC N IgM
0% (1%)
1% (15%)
5% (46%)
0% (0%)
99% (85%)
5% (4%)
0% (0%)
90% (49%)
VC S1 IgA
0% (5%)
0% (2%)
19% (74%)
0% (0%)
100% (98%
5% (0%)
0% (0%)
76% (21%)
VC S1 IgG
3% (4%)
0% (0%)
53% (90%)
0% (0%)
100% (100%)
3% (1%)
0% (0%)
42% (5%)
VC S1 IgM
0% (3%)
1% (8%)
6% (50%)
0% (0%)
99% (92%)
5% (3%)
0% (0%)
88% (45%)
VC S2 IgA
0% (4%)
0% (6%)
21% (77%)
0% (0%)
100% (94%
5% (1%)
0% (0%)
74% (18%)
VC S2 IgG
0% (3%)
0% (1%)
17% (59%)
0% (0%)
100% (99%
5% (3%)
0% (0%)
78% (36%)
VC S2 IgM
1% (4%)
2% (22%)
5% (50%)
0% (0%)
98% (78%)
4% (1%)
0% (0%)
90% (45%)
Measurement value
MG-RBD
Ground truth
True-negative
True-positive
Unknown
Cut-off
Manufacturer
Optimised

Suppl.Fig.9N

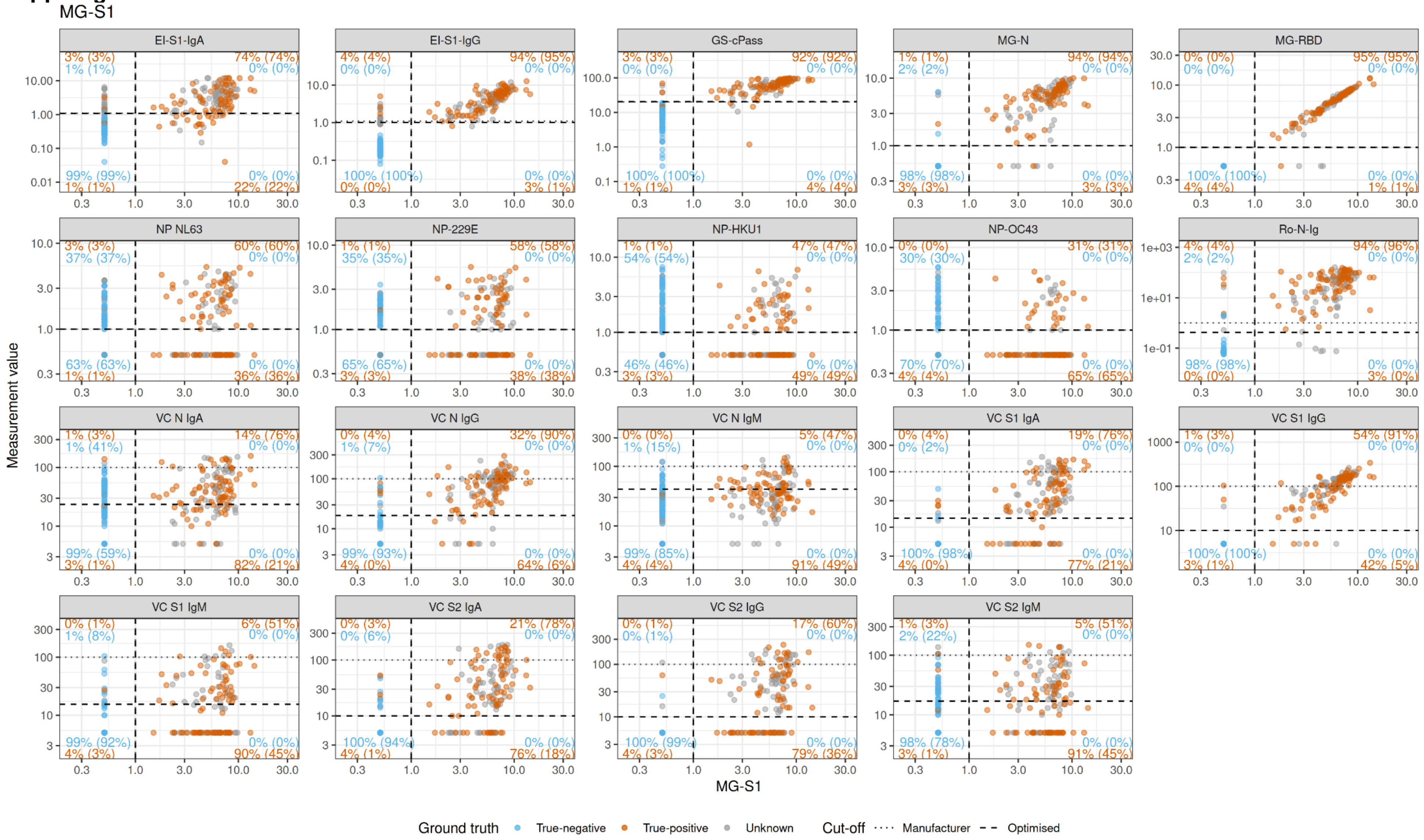

MG-S1
EI-S1-IgA
EI-S1-IgG
GS-cPass
MG-N
MG-RBD
NP NL63
NP-229E
NP-HKU1
NP-OC43
Ro-N-Ig
VC N IgA
VC N IgG
VC N IgM
VC S1 IgA
VC S1 IgG
VC S1 IgM
VC S2 IgA
VC S2 IgG
VC S2 IgM
Measurement value
MG-S1
Ground truth
True-negative
True-positive
Unknown
Cut-off
Manufacturer
Optimised

Supplemental Figure 9: Scatterplots of confirmatory tests vs confirmatory test

In blue the true-negatives, in orange the true-positives, in grey the values with unknown SARS-CoV-2 status. Black dotted and dashed lines represent the manufacturer's and the optimised positivity cut-offs, respectively. Orange/blue numbers give the percentages of true-positives/-negatives correctly detected by the tests using optimised cut-offs. The orange values above the dotted line represent the percentages of positive test results for the true-positives, the blue number below is the percentage of negatives in the true-negatives.

(A) Values obtained with NT
(B) Values obtained with GS-cPass
(C) Values obtained with VC-N-IgA
(D) Values obtained with VC-N-IgM
(E) Values obtained with VC-N-IgG
(F) Values obtained with VC-S1-IgA
(G) Values obtained with VC-S1-IgM
(H) Values obtained with VC-S1-IG
(I) Values obtained with VC-S2-IgA
(J) Values obtained with VC-S2-IgM
(K) Values obtained with VC-S2-IgG
(L) Values obtained with MG-N
(M) Values obtained with MG-RBD
(N) Values obtained with MG-S1

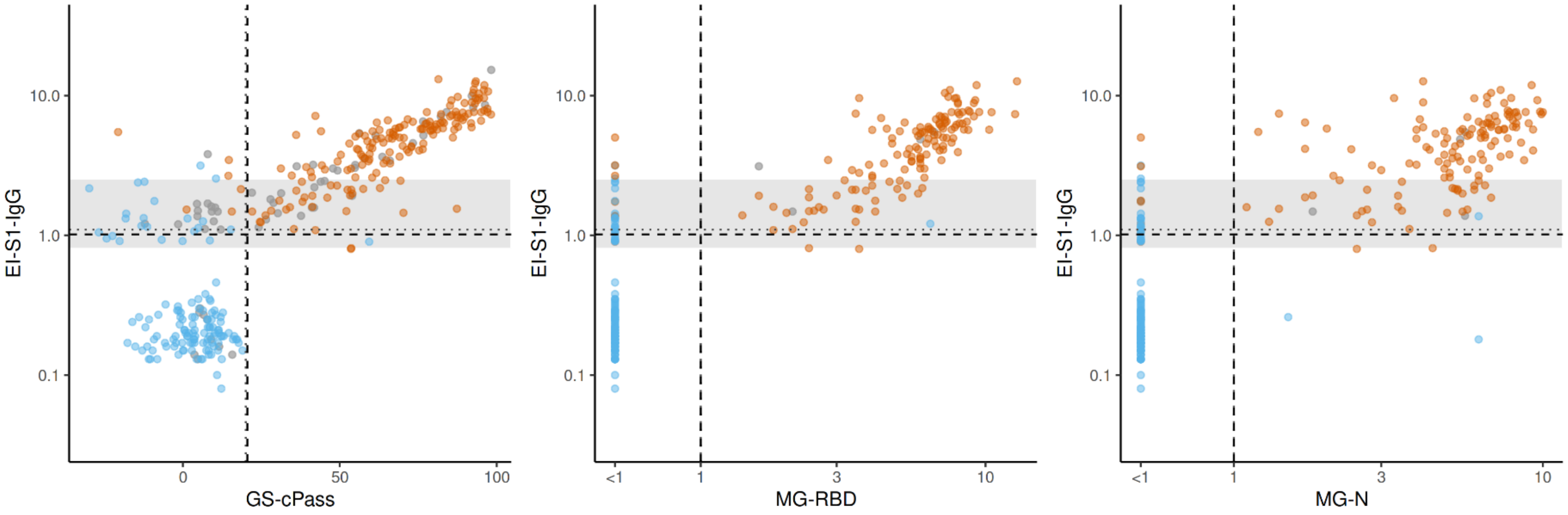

Supplemental Figure 10: Correlation between EI-S1-IgG with confirmatory tests GS-cPass, MG-RBD and MG-N. Visualization of the dataset uses an extended true positive definition, samples which measure positive in the other two confirmatory tests not under investigation were also defined as positive. By using stepwise optimization, test combinations were evaluated for performance. Better overall accuracy could be achieved by combining the tests in samples with raw values of the primary test in certain ranges (see Suppl. Table 5; visualized in grey above).

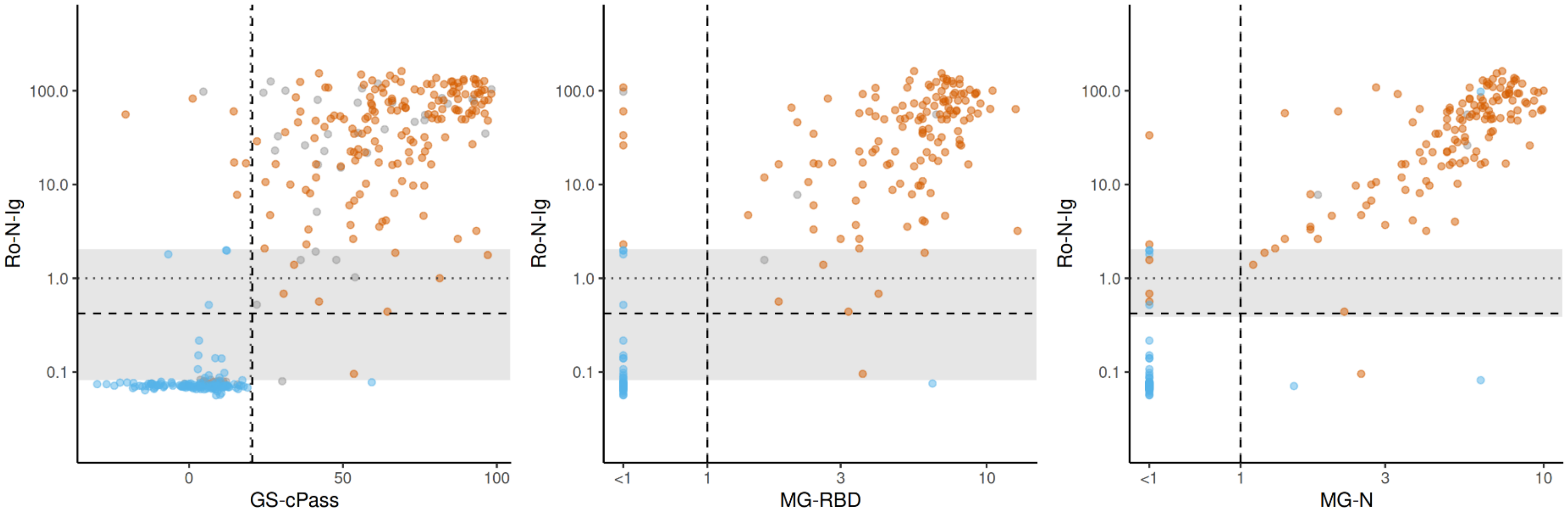

Supplemental Figure 11: Correlation between Ro-N-Ig with confirmatory tests GS-cPass, MG-RBD and MG-N. Visualization of the dataset uses an extended true positive definition, samples which measure positive in the other two confirmatory tests not under investigation were also defined as positive. By using stepwise optimization, test combinations were evaluated for performance. Better overall accuracy could be achieved by combining the tests in samples with raw values of the primary test in certain ranges (see Suppl. Table 5; visualized in grey above).
